# CHOICE-UTI: protocol for a multicentre randomised non-inferiority trial comparing single versus three daily doses of intravenous antibiotics prior to oral therapy for complicated urinary tract infection in children

**DOI:** 10.1136/bmjopen-2026-119766

**Published:** 2026-11-11

**Authors:** Laila F Ibrahim, Francesca Orsini, Amit Kochar, Simon Craig, Stuart Dalziel, Sarah McNab, Catherine Quinlan, Amanda Williams, Barry Scanlan, Meredith L Borland, Andrew Davidson, Ariel Mace, Scott Sypek, Li Huang, Franz E Babl, Penelope A Bryant

**Affiliations:** 1 Clinical Infections, Murdoch Children’s Research Institute, Parkville, Victoria, Australia; 2 Department of General Medicine, Royal Children’s Hospital Melbourne, Parkville, Victoria, Australia; 3 Department of Paediatrics, The University of Melbourne, Melbourne, Victoria, Australia; 4 Hospital in the Home, The Royal Children’s Hospital Melbourne, Melbourne, Victoria, Australia; 5 Clinical Epidemiology and Biostatistics Unit, Murdoch Children’s Research Institute, Parkville, Victoria, Australia; 6 Melbourne Children’s Trials Centre, Parkville, Victoria, Australia; 7 Paediatric Emergency, Women and Children’s Hospital Adelaide, Adelaide, South Australia, Australia; 8 Division of Acute Care Medicine, School of Medicine, College of Health, Adelaide University, Adelaide, South Australia, Australia; 9 Emergency Department, Monash Children's Hospital, Clayton, Victoria, Australia; 10 Clinical Sciences, Murdoch Children’s Research Institute, Parkville, Victoria, Australia; 11 Department of Paediatrics, Monash University, Melbourne, Victoria, Australia; 12 Department of Paediatrics, Child and Youth Health, The University of Auckland, Auckland, Auckland, New Zealand; 13 Department of Surgery, Child and Youth Health, The University of Auckland, Auckland, Auckland, New Zealand; 14 Children’s Emergency Department, Starship Children’s Health, Newmarket, Auckland, New Zealand; 15 Clinical Paediatrics, Murdoch Children’s Research Institute, Parkville, Victoria, Australia; 16 Kidney Regeneration, Murdoch Children's Research Institute, Parkville, Victoria, Australia; 17 Nephrology Department, Royal Children’s Hospital, Melbourne, Victoria, Australia; 18 Emergency Research, Murdoch Children's Research Institute, Parkville, Victoria, Australia; 19 Emergency Department, The Royal Children’s Hospital Melbourne, Melbourne, Victoria, Australia; 20 General Paediatric Department, Children’s Health Ireland, Dublin, Leinster, Ireland; 21 School of Medicine, University College Dublin School of Medicine, Dublin, Leinster, Ireland; 22 Emergency Department, Perth Children’s Hospital, Nedlands, Western Australia, Australia; 23 Divisions of Paediatrics and Emergency Medicine, The University of Western Australia Medical School, Perth, Western Australia, Australia; 24 Department of Anaesthesia, Royal Children's Hospital Melbourne, Parkville, Victoria, Australia; 25 Wesfarmers Centre of Vaccines and Infectious Diseases, The Kids Research Institute Australia, The University of Western Australia, Perth, Western Australia, Australia; 26 Department of General Paediatrics, Perth Children's Hospital, Nedlands, Western Australia, Australia; 27 Department of General Medicine, Women's and Children’s Hospital Adelaide, North Adelaide, South Australia, Australia; 28 Child Health Economics Unit, Centre for Health Policy, The University of Melbourne, Melbourne, Victoria, Australia; 29 Department of Critical Care, The University of Melbourne, Melbourne, Victoria, Australia; 30 Infectious Diseases, The Royal Children’s Hospital Melbourne, Melbourne, Victoria, Australia

**Keywords:** Paediatric infectious disease & immunisation, Antibiotics, Urinary tract infections

## Abstract

**Introduction:**

Children with urinary tract infection (UTI) frequently receive intravenous (IV) antibiotics when clinicians consider the infection to be complicated or when oral therapy is not considered appropriate. However, the optimal duration of IV therapy before switching to oral antibiotics in this population remains unknown. The CHOICE-UTI trial aims to determine whether a single dose of IV antibiotic followed by oral therapy is non-inferior to three daily doses of IV antibiotic followed by oral therapy for children with complicated UTI.

**Methods:**

This is a multicentre, open-label, non-inferiority randomised controlled trial conducted across emergency departments in Australia and New Zealand. Children aged 3 months to 18 years presenting with febrile UTI and at least one complicating feature for whom the treating clinician determines that IV antibiotics are required are eligible. Participants are randomised 1:1 to receive either one dose or three doses of once-daily IV antibiotics before switching to oral antibiotics, with both groups receiving a total of 7 days of therapy. The primary outcome is treatment failure at 72 hours after the first IV antibiotic dose, defined as persistence or development of fever, vomiting or rigors attributable to UTI. Secondary outcomes include readmission, antibiotic exposure, complications, recurrence of UTI and health-related quality of life. A total of 396 participants will be recruited.

**Ethics and dissemination:**

Ethics approval was obtained from the Royal Children’s Hospital Human Research Ethics Committee (HREC/72065/RCHM-2021). Results will be disseminated through peer-reviewed publications and scientific meetings, and participating families will receive a summary of the study findings.

**Trial registration number:**

NCT04876131.

**STRENGTHS AND LIMITATIONS OF THIS STUDY**

- This is a multicentre clinical trial with clearly defined widely applicable inclusion criteria using clinical features and a low-cost urine dipstick test.
- This is the first randomised trial of a single IV dose versus a multi-dose IV course before oral switch for complicated UTIs.
- The primary outcome is patient and family-centred, based on parent-reported symptoms.
- Quality of life is measured alongside a formal health economic analysis.
- This is an unblinded study which may result in clinician bias, however is mitigated by using a parent-reported primary outcome.

## Introduction

 Randomised trials have demonstrated that most children with urinary tract infections (UTI), even with fever, can be successfully treated with oral antibiotics alone.^1–4^ Despite this, a substantial proportion of children continue to receive intravenous (IV) antibiotics in hospital. In Australia, UTIs account for 12% of preventable hospitalisations across all age groups, including in children.^5 6^ These hospitalised children are often considered to have a ‘complicated UTI’. However, there is no universally accepted definition of complicated UTI in paediatrics. Existing definitions incorporate a range of factors, including anatomical abnormalities of the urinary tract, recurrent infections, host comorbidities and clinical severity.^6–9^ In practice, the decision to initiate IV antibiotics is largely driven by clinician judgement when one or more of these features are present, yet evidence to guide both this decision and the optimal duration of IV therapy remains limited.^10^

The rationale for initial IV antibiotics is to achieve rapid, high drug concentrations at the site of infection, particularly when the child is systemically unwell or when renal parenchymal involvement is suspected.^11 12^ Children with these complicating features of UTI have been excluded from previous trials and Cochrane reviews, resulting in a lack of guidance on the appropriate duration of IV antibiotic treatment and timing for switching to the oral route.^13–15^ The only clinical trial investigating duration of IV antibiotics for UTI, conducted over two decades ago, compared 3 versus 10 days of IV antibiotics and found no significant difference, supporting a shorter duration.^16^ Without any newer evidence, international paediatric guidelines either give no duration for IV antibiotics or recommend reassessment for oral switch within 48 hours in most cases of pyelonephritis.^17–19^ Despite guidelines recommending early oral switch, hospitalised children receive, on average, 3 days of IV antibiotics before transitioning to oral therapy.^20 21^ Taken together, the optimal duration of IV antibiotic therapy before transition to oral treatment in children with complicated UTI has never been prospectively evaluated.

Observational studies in emergency departments suggest that when IV antibiotics are deemed clinically necessary, a single once-daily dose can be used as a pragmatic approach.^7 22^ Unnecessary prolonged IV therapy exposes children to the risks of IV cannulation, extended hospitalisation, and greater cumulative antibiotic burden, all of which are avoidable if a shorter course proves equally effective.^23 24^ Previous studies suggest that a single IV dose may be comparable to a multi-dose course across a broad group of children, including those with complicated features, yet no prospective trial has evaluated this directly.^7 25–27^ We propose the first randomised controlled trial to investigate whether a single IV antibiotic dose is as effective as the standard three-dose regimen before switching to oral antibiotics for a total 7-day course in children presenting to the ED with a complicated UTI. A non-inferiority result would support a shorter IV course and inform evidence-based guidelines for complicated UTI management in children, reducing unnecessary hospitalisation and antibiotic exposure.

## Methods

### Trial design and study setting

This study is an open label, multicentre, non-inferiority randomised controlled trial. It employs a two-arm, non-inferiority approach with parallel arms and 1:1 allocation to single dose or 3 doses of a once daily IV antibiotic prior to switch to oral antibiotics for a total of 7 days, conducted at seven tertiary-level hospitals across Australia and New Zealand. Recruitment commenced in January 2022 and is expected to be conducted for 5 years. Participating sites were activated sequentially over approximately 12 months as governance approvals were obtained.

### Patient and public involvement in the trial design

Patients enrolled in two previous studies informed the study question and the eligibility criteria.^10^ A parent consumer advisor with lived experience as the parent of a child with recurrent hospital admissions for UTI, was involved in the development of the study design, specifically regarding the appropriateness of the trial intervention and the primary outcome. The primary outcome was chosen as a measure that was important to children and their families rather than a microbiological, imaging or laboratory-based outcome. Ongoing parental consumer involvement includes periodic review of recruitment processes and follow-up procedures.

There is no universally accepted definition of complicated urinary tract infection in children. Definitions in the literature include a range of clinical, anatomical and host factors. In this trial, complicated UTI is operationally defined as a febrile UTI in which the treating clinician determines that IV antibiotics are indicated due to clinical severity or risk factors such as recurrent infection or known urological abnormalities. This pragmatic definition reflects real-world decision making in emergency departments and aligns with previous observational studies of parenteral therapy for paediatric UTI.

### Eligibility criteria

Children aged 3 months (corrected age) to 18 years presenting to the emergency department (ED) with suspected complicated UTI will be eligible if they have fever (reported or measured≥38.0 °C) and at least one complicating feature: including a history of previous UTI, known urological abnormality (of any severity), vomiting, rigors, or documented tachycardia above age-appropriate reference ranges. Based on these, the treating ED clinician will determine indication for IV antibiotics. A urine specimen must be collected before IV antibiotic administration by clean catch, catheterisation, or suprapubic aspirate, with evidence of UTI on dipstick or microscopy (leucocyte esterase>1+or nitrite positivity or≥10 white blood cells per high-power field, or bacteriuria). UTI will be confirmed subsequently as follows: 1) growth of a dominant uropathogen≥10⁵ colony-forming units/mL from a clean-catch specimen or≥10⁴ colony-forming units/mL from a catheter specimen or from a suprapubic aspirate; 2) radiological findings consistent with upper urinary tract infection, eg, renal parenchymal abnormalities on ultrasound (imaging is performed only when clinically indicated and is not required for trial participation); and/or 3) the treating clinician has diagnosed the patient as having a UTI and treated as such with a full course of antibiotics (anticipated to be the minority).

### Exclusion criteria

Children will be excluded if they have presumed sepsis requiring>20 mL/kg fluid resuscitation or inotropic support; a concurrent condition requiring more than one dose of IV antibiotics (eg, meningitis); chronic kidney disease, kidney transplant, unrepaired posterior urethral valves, indwelling ureteric stent, or suspected renal abscess; allergy to all study antibiotics (gentamicin, ceftriaxone, amikacin); no suitable oral antibiotic option based on known resistance; prior IV antibiotics for the same UTI episode; clinician intent to administer only a single IV dose from the outset; previous enrolment in the CHOICE-UTI trial; UTI within the preceding 2 weeks; or pregnancy.

### Recruitment, participant screening and consent

During ED assessment, patients with fever and suspected complicated UTI requiring IV antibiotics will be screened. If eligible, patients and their guardians will be approached for informed consent (online supplemental material 1). Where appropriate, children will be involved in the consent process in accordance with their age and developmental capacity, in accordance with local ethical guidelines.

### Allocation and randomisation

After informed consent, participants will be randomised 1:1 to either treatment arm using secure web-based randomisation via REDCap, with emergency sealed-envelope backup during system downtime. The randomisation schedule was prepared by an independent statistician using randomly permuted blocks of variable size, stratified by age (<1 year vs≥1 year), number of complicating factors (≤3 vs ≥4), and recruitment site. Randomisation will be undertaken by trained study staff, and both clinicians and participants will be aware of treatment allocation. Enrolment and randomisation will occur before the first once-daily IV antibiotic dose; to avoid treatment delay in the emergency department, randomisation within 2 hours of the initial dose is permitted, with enrolment beyond this window allowed only when research staff are unavailable or clinical workload precludes recruitment. The time between the first IV antibiotic dose and trial enrolment will be recorded and reported in the trial results.

### Interventions

All participating hospitals use a once daily IV antibiotic for UTI treatment. The standard options include gentamicin or ceftriaxone, with amikacin reserved for cases of known gentamicin resistance. IV antibiotic choice follows each hospital’s empirical guidelines for UTI, which are based on local bacterial susceptibility patterns. This is to ensure the inclusivity of patients and generalisability of outcomes, as this is a study of antibiotic route, not choice. Some hospitals additionally empirically cover for the possibility of *Enterococcus* spp with multiple-daily dosing of IV penicillin or amoxicillin. When indicated, the study antibiotic is switched to the antibiotic to which the organism is susceptible, as per standard clinical practice to ensure effective treatment.

Participants will be randomised to receive either 1 dose of daily IV antibiotics with switch to oral antibiotics or 3 doses of daily IV antibiotics and then switch to oral antibiotics with both arms receiving a total of 7 days’ antibiotics (figure 1). After the first dose of IV antibiotic, irrespective of the study arm, the child will either be admitted to an inpatient ward or, where available, transferred to the Hospital-in-the-Home (HITH) programme. If the IV cannula stops working or is unable to be sited, the treating clinician may administer the antibiotic dose intramuscularly. Treating clinicians may extend IV therapy beyond the assigned duration if deemed clinically necessary. Such deviations will be recorded prospectively and included in secondary analyses examining total IV antibiotic exposure. To minimise protocol deviations, ward clinicians will be provided with study-specific information outlining the trial rationale and protocol, including guidance that persistent fever in isolation does not indicate a need to continue IV antibiotics when other clinical features support transition to oral therapy or monitoring via the Hospital-in-the-Home programme.

**Figure 1:**
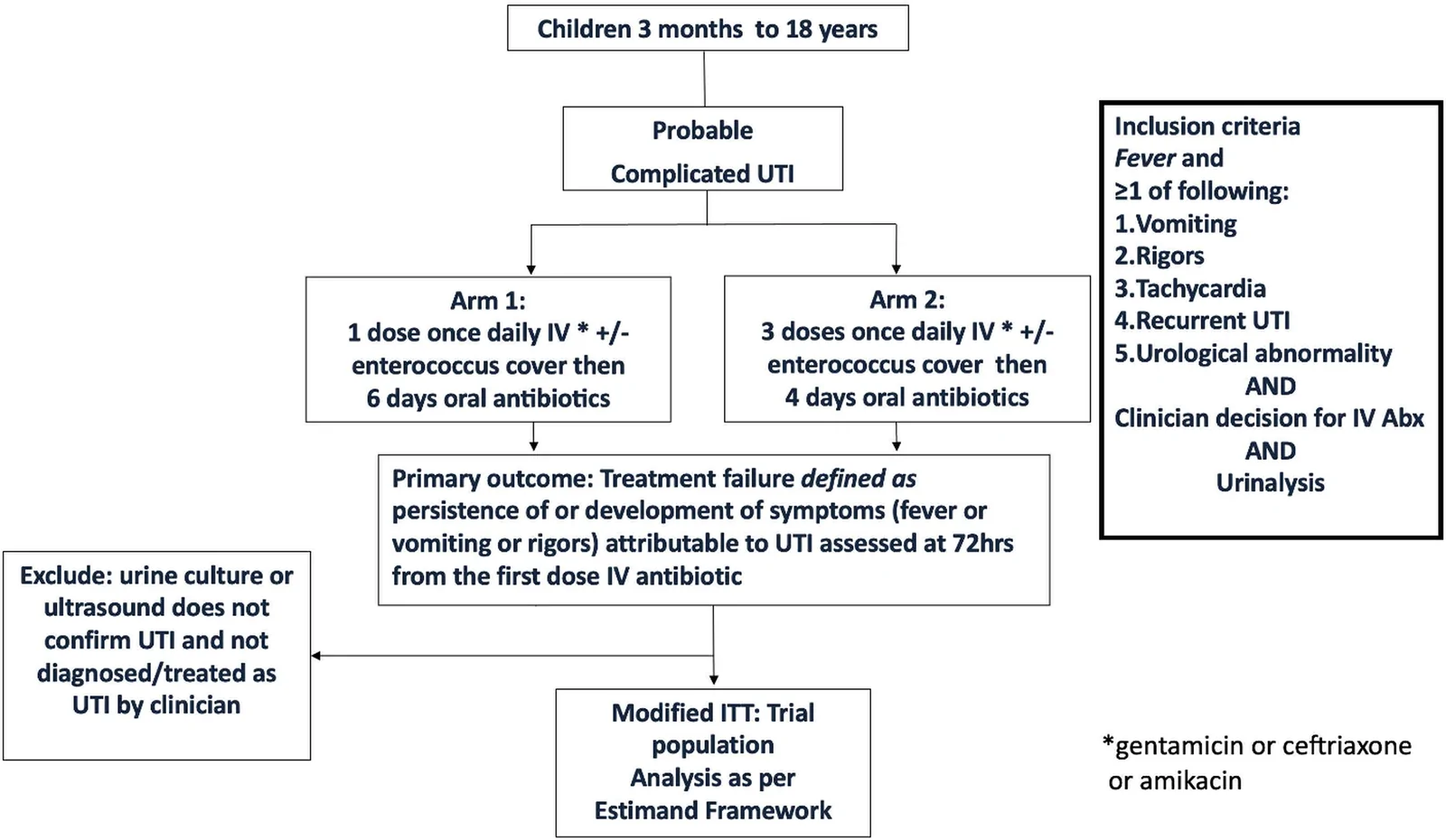
Trial intervention schema. Participants randomised to the one-dose arm receive a single daily dose of intravenous (IV) antibiotic before switching to oral antibiotics; participants randomised to the 3-dose arm receive three daily doses of IV antibiotic before switching to oral antibiotics. Both arms receive a total of 7 days of antibiotic therapy. Abx, antibiotics, ITT, intention-to-treat; UTI, urinary tract infection.

In both arms, within 18 to 24 hours of the last IV dose, the participant will switch to oral antibiotics for the remainder of their 7-day antibiotic course. Oral antibiotics will be cephalexin or trimethoprim and/or sulfamethoxazole, depending on availability at participating sites and urinary pathogen susceptibility testing. (figure 2) If a child vomits within 30 min of receiving the oral antibiotic, as per routine clinical advice a repeat dose is advised.

**Figure 2:**
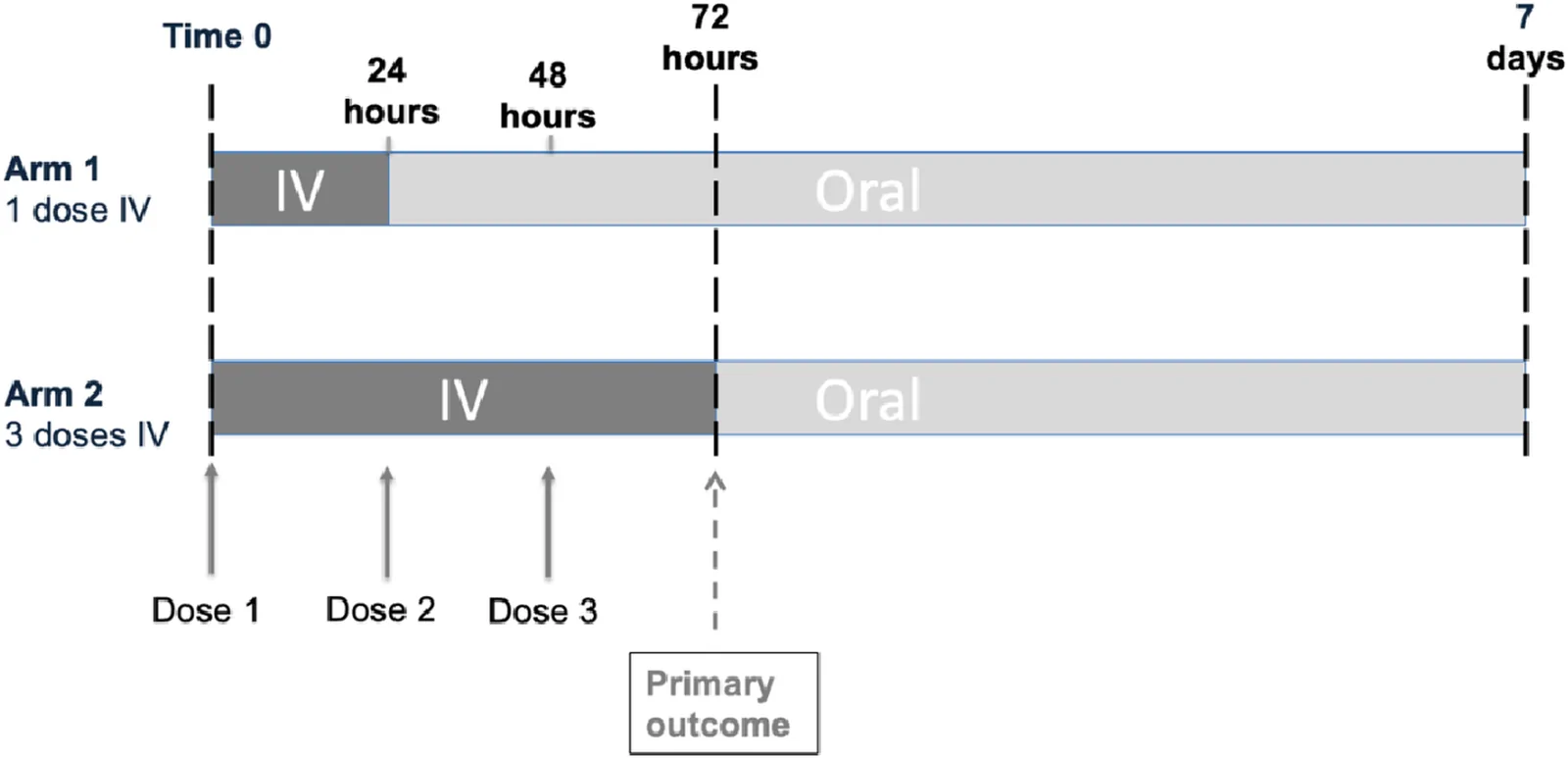
Duration of intravenous and oral antibiotics in each study arm.

Antibiotic resistance patterns and pathogen susceptibility will be recorded and reported, including resistance to third-generation cephalosporins and aminoglycosides.

### Outcomes

The primary outcome is defined as treatment failure, measured at 72 hours following the first IV antibiotic dose. Treatment failure is defined as persistence of baseline symptoms (fever, vomiting or rigors) or development of new symptoms after resolution (fever, vomiting, or rigours) attributable to UTI, assessed at least 6 hours after the last antipyretic. In the anticipated small number of cases where it is unclear whether new symptoms are attributable to UTI, a de-identified clinical history, examination and investigations findings will be provided to an independent blinded clinician who will adjudicate. Secondary outcomes include readmission within 14 days and 1 month, time to resolution of fever, total duration of IV antibiotics and oral antibiotics, recurrence of UTI within 14 days and 1 month, complications (eg, kidney abscess, meningitis and sepsis), adverse drug reactions, quality of life indicators, cost-effectiveness and identification of kidney abnormalities on ultrasound (if performed).

#### Data collection and management

Participant demographic and clinical data will be extracted from medical records (table 1). Follow-up will occur at 72 hours, 14 days and 1 month. During the first 72 hours, parents or guardians will prospectively record fever, vomiting and rigors using a provided symptom diary and hospital-grade axillary thermometer, with symptoms recorded three times daily. At day 14, parents or guardians will report work absenteeism and illness-related out-of-pocket costs. Child health-related quality of life will be assessed using the Child Health Utility 9D (CHU9D), completed by children aged 7 to 17 years or by proxy for younger children, and parent quality of life will be assessed using the EuroQol 5-Dimension instrument. Data will be captured in REDCap and analysed by the Chief Principal Investigator with oversight from the trial statistician, both of whom will remain blinded to treatment allocation until analysis completion.

**Table 1:** Study schedule

	Day 1	Day 2	Day 3	Day 4	Days 5–7	Day 14	1 month
Time point*	ED presentation (first dose of IV antibiotic)	24 hours	48 hours	72 hours			
Enrolment:							
Eligibility screen	X						
Informed consent	X						
Allocation to intervention	X						
Interventions:							
Arm 1: one dose	IV	Oral	Oral	Oral	Oral		
Arm 2: three doses	IV	IV	IV	Oral	Oral		
Assessments:							
Demographic information†	X						
Clinical assessment ‡	X						
Parental assessment daily§	X	X	X	X			
Parental cost data¶						X	
Primary outcome**				X			
Clinical assessment – other criteria††	X	X	X	X			
Day 14 review‡‡						X	
QoL survey§§	X			X		X	
Final review ¶¶							X

* Timepoint refers to the time lapsed from the initial first dose of IV antibiotic.

† Patient demographic information will be collected from the patient’s clinical notes.

‡ This will include presence of: lethargy, abdominal pain, flank pain/tenderness, dysfunctional voiding, dysuria, offensive urine, gross haematuria, frequency, suprapubic tenderness, antibiotic use (prophylaxis), known resistant organisms, method of urine collection in ED will be documented.

§ Daily parental assessment: parent/guardians will be asked to prospectively record presence of fever, vomiting, rigors in a diary which will be provided at enrolment.

¶ Parental cost data collection: parent/guardians will be asked to report in the previous 14 days since the first dose of antibiotic—leave taken for paid and unpaid work, extra expenditure related to the child’s illness such as medication, transport, accommodation, childcare, food, collected at Day 14.

** Primary outcome: presence of fever, vomiting, rigors reported by parents/guardians.

†† Other clinical information including: adverse events and complications.

‡‡ The 14-day review for recurrence of UTI symptoms or hospital admission for UTI.

§§ The Child Health Utility 9D and the EuroQol 5 Dimension will be administered to measure child and parent quality of life.

¶¶ The 1-month review for recurrence of UTI or hospital admission for UTI.

#### Statistical methods

Primary and secondary outcomes will be compared between treatment arms in a modified intention-to-treat population comprising all randomised participants with a confirmed diagnosis of UTI, defined by urine culture, radiological evidence and/or full treatment as UTI. Participants randomised but subsequently found to be ineligible (eg, presumed or confirmed alternate diagnosis) will be replaced and excluded from analysis, as confirmation of UTI is not available until 48 to 72 hours after baseline sample collection.

Intercurrent events will be handled using the estimand framework, described in the International Council for Harmonisation guideline.^28^ Treatment crossover (patients switch from their assigned arm to the other arm, eg, a participant in the one dose arm continues to receive additional IV doses or a participant in the three doses arm stopping after the first IV dose) will be addressed using a hypothetical strategy for all outcomes by setting outcomes to missing following this intercurrent event and using multiple imputation to recover the missing values. A supplementary treatment policy analysis will be performed, retaining outcomes after crossover and analysing participants according to their randomised group.

The primary outcome, treatment failure 72 hours after the first dose of IV, will be compared between treatment arms using a risk difference and its 95% CI estimated using a generalised linear model with binomial family and identity link function, adjusted for randomisation strata as a covariate. For one dose to be non-inferior to three doses, the upper limit of the two-sided 95% CI of the risk difference must be less than 10 percentage points in both analyses. Secondary outcomes will be compared between treatment arms using appropriate generalised linear models according to the outcome type, with corresponding effect measures (eg, mean differences, risk differences) and 95% CIs reported. Supplementary analyses will be performed controlling for any baseline covariates that are likely to be directly related to the outcome. Treatment effect modification will be assessed by testing for interactions between treatment arm and the following variables: age (<1 year vs≥1 year), and number of complicating factors at presentation in the emergency department (≤3 vs ≥4 factors). Pre-planned subgroup analyses for the primary outcome will be conducted for age (<1 year vs≥1 year) and number of complicating factors (≤3 vs ≥4 factors) and presence of underlying urological abnormalities. A formal health economic analysis will be undertaken.

### Sample size estimation

Robust evidence for 72-hour clinical failure after a single IV dose is limited; given the high efficacy of oral therapy, we expect rates to be similar to 3 days of IV antibiotics. We prespecified a non-inferiority margin of 10 percentage points above the 72-hour failure proportion in the three-dose group, assumed at 5%. The margin was agreed a priori by clinician investigators across specialties, informed by consumer input and prior UTI and paediatric infection trials using margins of 10% or greater. Under this framework, one dose will be deemed non-inferior if the upper bound of the two-sided 95% CI for the risk difference is less than 10 percentage points. Assuming a 7% failure rate in the one-dose arm and 5% in the three-dose arm, with 85% power and α=0.05, a sample of 316 children is required; a 20% rate of loss to follow-up or protocol deviations is expected, therefore 396 children will be recruited, ie, 198 in each arm.

#### Independent safety and data monitoring committee

An independent safety and data monitoring committee (ISDMC) has been established. The ISDMC consists of two independent clinicians (paediatrician/emergency physician and paediatric nephrologist) and an independent biostatistician who, collectively, have experience in the management of paediatric patients with UTI and in the conduct and monitoring of randomised controlled trials. The ISDMC will function independently of all other individuals and bodies associated with the conduct of the study. The ISDMC will review all data by treatment arm every six to twelve months from trial commencement.

As of August 2026, 290 children have been recruited across seven hospitals in Australia and New Zealand. The trial remains open to recruitment. This protocol is reported in accordance with the SPIRIT (Standard Protocol Items: Recommendations for Interventional Trials) guidelines^29^ and was registered in the clinical trials registry before participant recruitment started (ClinicalTrials.gov ID: NCT04876131).

#### Ethics and dissemination

Ethics approval was obtained from the Royal Children’s Hospital Human Research Ethics Committee (HREC/72065/RCHM-2021). Since the protocol was first approved, amendments were made as follows, to: simplify the eligibility criteria to align with a busy emergency setting; broaden the subsequent confirmation of UTI diagnosis in enrolled patients to include – in addition to urine culture positivity – children with ultrasonographic evidence of UTI, and/or children whose treating clinicians diagnosed and treated them fully as having a UTI; reduce the sample size inflation from 30% to 20% as the rates of dropout and deviations were not as high as initially adjusted for (online supplemental material 2), trial protocol). Results will be disseminated through peer-reviewed publications and scientific meetings, and participating families will receive a summary of the study findings.

## Discussion

The optimal duration of IV antibiotic therapy for children with complicated UTI is unknown. The absence of prior trials likely reflects challenges in defining ‘complicated UTI’, for which no universally accepted definition exists. Definitions used in clinical practice and research incorporate a range of factors including anatomical abnormalities of the urinary tract, recurrent infection, host comorbidities and markers of clinical severity.^30^ Recent guidance from the European Society for Paediatric Infectious Diseases similarly highlights the heterogeneous nature of this population and emphasises the importance of clinical context when determining management.^31^ This randomised controlled trial aims to provide much-needed evidence for IV duration, and critical for ED clinicians, for the additional question of whether a child needs to be admitted to hospital for subsequent doses of IV antibiotics after the first IV dose is administered in ED (i.e. randomisation happens at the initial dose, not 24 hours later).

In the present trial, the inclusion criteria reflect real-world clinical decision making. Children are eligible when they present with febrile UTI and at least one complicating feature and the treating clinician determines that IV antibiotics are required. While this approach captures a heterogeneous population, it reflects the spectrum of children commonly presenting to ED for whom there is no guidance on IV duration, and improves the external validity of the study. Prespecified subgroup analyses will explore treatment effects in clinically relevant populations including those with urological abnormalities.

Selecting a primary outcome that balances scientific rigour with clinical and caregiver relevance presents a significant challenge. Older trials used delayed imaging as the primary outcome, while latterly, clinical or microbiological cure have been used.^21^ A recent suggestion for the primary outcome for UTI studies was to combine clinical cure (defined as defervescence) and microbiological evaluation at 48 to 72 hours after starting treatment.^15^ We chose a clinical primary outcome of the presence of fever at 72 hours, with the additional assessment of other systemic symptoms, namely vomiting and rigours. Measuring presence of fever, vomiting and rigours speaks directly to a child ‘clinically improving’. While some children may remain symptomatic at 72 hours without representing treatment failure, this timepoint reflects a clinically meaningful decision point for escalation or modification of therapy. Importantly, we opted not to collect urinary microbiological clearance data after starting treatment due partly to the challenges of obtaining urine samples from children, and partly because the child will not have finished treatment at the point of interest (i.e. the current usual time of switching from IV to oral antibiotics). Proof of microbiological cure is not routine clinical practice in Australia and New Zealand and was not deemed ethically justifiable given logistical difficulties, as it would not add value to the clinical outcomes or inform the necessary duration of IV antibiotics.^32^

Another challenge for designing this trial was the non-inferiority margin. Approximately 5% of children with complicated UTI treated with 3 days of IV treatment are expected to take longer than 72 hours to clinically improve, i.e. fail the primary outcome of this study. Given the potential benefits of reducing IV antibiotics exposure and the benefit of earlier hospital discharge a wider non-inferiority margin was deemed appropriate.

Another potential challenge is adherence to the assigned treatment duration. In clinical practice, clinicians may extend or shorten IV therapy based on the child’s clinical course. To address this, clinicians with patients participating in the trial received information regarding the study protocol, and deviations from the assigned treatment arm will be recorded prospectively. This will allow transparent reporting of adherence and enable exploration of factors associated with protocol deviations. Additionally, at most sites, clinicians will be able to refer to the HITH programme to either continue IV antibiotics at home or for further clinical monitoring of children on oral therapy.

### Strengths and limitations

This randomised trial has several strengths. It is a bi-national multicentre randomised controlled trial conducted across multiple healthcare systems, enhancing generalisability. The pragmatic design reflects routine clinical practice, including the use of local empirical antibiotic guidelines. In addition, outcomes were selected with input from families and consumer representatives to ensure that the study addresses clinically meaningful questions.^33^ Despite its strengths, this trial also presents several limitations. This includes the heterogeneity of presentation and severity, a challenge shared with previous work.^34^ However, prespecified subgroup analyses, including children with underlying urological abnormalities, will provide additional insights into treatment response in clinically relevant populations.

In conclusion, this multicentre trial addresses a critical gap in the management of paediatric complicated UTI by aiming to determine the necessary duration of IV antibiotics prior to oral switch. By incorporating healthcare institutions’ own empirical antibiotic choice guidelines, robust sample size, and inclusion of input from a broad range of stakeholders, the outcomes of this trial are poised to transform patient care, inform resource allocation, and set new benchmarks for antimicrobial stewardship in the management of complicated UTI.

## Supplementary material

10.1136/bmjopen-2026-119766 online supplemental file 1

10.1136/bmjopen-2026-119766 online supplemental file 2
