# Supplementary material for: CHOICE-UTI: protocol for a multicentre randomised non-inferiority trial comparing single versus three daily doses of intravenous antibiotics prior to oral therapy for complicated urinary tract infection in children
Source: BMJ Open. 2026 Nov 11;16(11):e119766. doi: 10.1136/bmjopen-2026-119766 (PMC13756224; doi:10.1136/bmjopen-2026-119766)
Supplement: Supplementary file 1 [file bmjopen-2026-119766.reviewer_comments.pdf]

PEER REVIEW HISTORY

BMJ Open publishes all reviews undertaken for accepted manuscripts. Reviewers are asked to complete a checklist review form and are provided with free text boxes to elaborate on their assessment. These free text comments are reproduced below.

ARTICLE DETAILS

Title (Provisional)

CHOICE-UTI: protocol for a multicentre randomised non-inferiority trial comparing one versus three doses of intravenous antibiotics prior to oral therapy for complicated urinary tract infection in children

Authors

Ibrahim, Laila; Orsini, Francesca; Kochar, Amit; Craig, Simon; Dalziel, Stuart; McNab, Sarah; Quinlan, Catherine; Williams, Amanda; Scanlan, Barry; Borland, Meredith L; Davidson, Andrew; Mace, Ariel; Sypek, Scott; Huang, Li; Babl, Franz E; Bryant, Penelope

---

VERSION 1 - REVIEW

**Reviewer**	**1**
**Name**	**Carlton, Edward**
**Affiliation**	**University of Bristol, Translational Health Sciences, Southmead Hospital Learning and Research**
**Date**	**23-Apr-2026**
**COI**	**None**

I always find it hard to review trial protocols as there is a limited amount that can be changed (especially given recruitment here is well underway). That said this is an excellent trial and the authors should be congratulated for writing up an excellent protocol and indeed the excellent recruitment they have achieved to date.

My minor comments are around improving clarity in the protocol:

1. The primary endpoint choice is patient centred and an excellent choice. I do think further clarity is required around how this is adjudicated, given the subjective nature of some elements of the composite outcome (eg vomiting attributable to UTI). How exactly would the blinded adjudicator judge this? Will they be provided with a clinical narrative? How will blinding be maintained within this narrative? Is one reviewer sufficient given the importance of the primary?

2. I note recruitment is well underway. Can the authors declare whether any significant changes in the design/protocol have been made since 2022?

3. I can see why it is being done but further justification of post randomisation exclusion of subsequent negative urine culture participants is required. I am never a fan of this approach in pragmatic trials. Further to this are these the "protocol violations" referred to in the sample size section-this could all be a bit clearer.

4. I am intrigued as to why formal health economic analysis is not being undertaken here (especially as you are collecting data pertaining to this). Could you mention what your plans are for health ec? My feeling is this would be needed to inform guideline change should you demonstrate non-inferiority.

Well done to the investigators in getting to this stage. A much needed and internationally important trial.

VERSION 1 - AUTHOR RESPONSE

Reviewer 1 — Dr Edward Carlton, University of Bristol

> *"I always find it hard to review trial protocols as there is a limited amount that can be changed (especially given recruitment here is well underway). That said this is an excellent trial and the authors should be congratulated for writing up an excellent protocol and indeed the excellent recruitment they have achieved to date. My minor comments are around improving clarity in the protocol."*

Comment R1.1 — Adjudication of the primary outcome

> *The primary endpoint choice is patient centred and an excellent choice. Further clarity is required around how this is adjudicated, given the subjective nature of some elements of the composite outcome (e.g. vomiting attributable to UTI). How exactly would the blinded adjudicator judge this? Will they be provided with a clinical narrative? How will blinding be maintained within this narrative? Is one reviewer sufficient given the importance of the primary?*

Response: We believe the number of participants where it is unclear whether new symptoms are attributable to UTI, will be very small. A de-identified clinical history, examination and investigations findings will be provided to an independent blinded clinician who will adjudicate. Given the anticipated small number of such cases, a single independent blinded adjudicator was considered sufficient.

Manuscript change: The following changes have been made in Methods, under subheading 'Outcomes': In the anticipated small number of cases where it is unclear whether new symptoms are attributable to UTI, a de-identified clinical history, examination and investigations findings will be provided to an independent blinded clinician who will adjudicate.

Comment R1.2 — Protocol changes since 2022

> *I note recruitment is well underway. Can the authors declare whether any significant changes in the design/protocol have been made since 2022?*

Response: See above under query E4.

Manuscript change: See above under query E4.

Comment R1.3 — Post-randomisation exclusion of negative urine culture participants

> *I can see why it is being done but further justification of post-randomisation exclusion of subsequent negative urine culture participants is required. I am never a fan of this approach in pragmatic trials. Further to this, are these the "protocol violations" referred to in the sample size section — this could all be a bit clearer.*

Response: We appreciate the reviewer highlighting this as we had discussed this at length at the outset, due to not wanting to conduct a trial where the easy criticism was whether the patients even had a UTI. We have therefore taken this helpful and supportive feedback back to the Trial Steering group. We have consensus agreement with the reviewer, both philosophically and since this does not change any patients we have already enrolled. It simply enables the trial to include the 20 already-enrolled patients treated fully as having a UTI by their clinicians, that we would have replaced under the original protocol due to lack of microbiological/radiological confirmation.. We have revised the subsequent confirmation of UTI diagnosis. Thank you for this valuable suggestion.

This also clarifies that these are not the patients who deviate from the protocol.

Manuscript change: Under Methods, subheading 'Eligibility criteria'**:** and/or if the treating clinician has diagnosed a patient as having a UTI and treated as such with a full course of antibiotics (anticipated to be the minority).

Comment R1.4 — Health economic analysis

> *I am intrigued as to why formal health economic analysis is not being undertaken here (especially as you are collecting data pertaining to this). Could you mention what your plans are for health economics? My feeling is this would be needed to inform guideline change should you demonstrate non-inferiority.*

Response: We are certainly conducting a formal health economic analysis, and this was an oversight in not clearly stating this.. We agree that this will be key to influence policy regardless of the outcome and we have two health economists as part of our team. Our plan and experience is that a health economic analysis is a separate body of work and the publication which will require its own aims and methodology.

Manuscript change: We have added 'A formal health economic analysis will be undertaken' under the 'Statistical methods' for clarity.

> *"Well done to the investigators in getting to this stage. A much needed and internationally important trial."*

Thank you (seriously) for this encouragement. It has been a hard trial to do, but we're slowly getting there and every piece of positive feedback helps.

VERSION 2 - REVIEW

Reviewer 1

Name Carlton, Edward

Affiliation University of Bristol, Translational Health Sciences, Southmead Hospital Learning and Research

Date **15-Sep-2026**

COI

Really impressed you took on board my feedback around post randomisation exclusions and have taken this to your steering committee and made changes to the protocol. A positive change in my view.

I wish you all the best on completing this excellent trial.
