# Supplementary material for: CHOICE-UTI: protocol for a multicentre randomised non-inferiority trial comparing single versus three daily doses of intravenous antibiotics prior to oral therapy for complicated urinary tract infection in children
Source: BMJ Open. 2026 Nov 11;16(11):e119766. doi: 10.1136/bmjopen-2026-119766 (PMC13756224; doi:10.1136/bmjopen-2026-119766)
Supplement: Supplementary file 2 [file bmjopen-16-11-s001.docx]

| **HREC Reference Number:** | 72065 | | |
| --- | --- | --- | --- |
| **Project title:** | Clinical efficacy of single dose (daily) IV antibiotics followed by 2 days oral antibiotics compared to 3 doses (daily) IV antibiotics for children with complicated urinary tract infections: a multicentre randomised trial | | |
| **Project Sponsor** | Murdoch Children’s Research Institute | | |
| **Co-ordinating Principal Investigator and Principal Investigator:** | Dr Laila Ibrahim, Paediatrician RCH, Clinician Scientist-Fellow MCRI and [Insert site Principal Investigator of site here] | | |
| **Location** | [Insert location here] | | |
| **Version Number:** | 7 | **Version Date:** | 6/6/2024 |

We may have asked you already for your verbal consent, we are now asking for your written consent.

Thank you for taking the time to read this **Parent and/or Guardian Information Statement and Consent Form**. We would like to invite your child to take part in a research project that is explained in this form.

This form is 8 pages long. Please make sure you have all the pages.

**What is an Information Statement and Consent Form?**

An Information and Consent Form tells you about the research project. It explains what the research project involves. This information is to help you decide whether or not you would like your child to take part in the research. Please read it carefully. Ask questions about anything you don’t understand or want to know more about. Before deciding whether or not to take part, you might want to talk about it with a relative, friend or your child’s local doctor.

Before you decide if you want your child to take part or not, you can ask us any questions you have about the project. You may want to talk about the project with your family, friends or health care worker.

**Taking part in the research project is up to you**

It is your choice whether or not your child takes part in the research project. You do not have to agree if you do not want to. If you decide you do not want to take part, it will not affect the treatment and care your child receives at [Insert site here].

**Signing the form**

If you want your child to take part in the research, please sign the consent form at the end of this document. By signing the form you are telling us that you:

- Understand what you have read
- Had a chance to ask questions and received satisfactory answers
- Consent to taking part in the project.
- Consent for your child to take part in this research project
- Consent for your child to have the tests and treatments that are described.
- Consent to the use of your child’s personal and health information as described.

We will give you a copy of this form to keep.

1. **What is the research project about?**

Urinary Tract Infections (UTI) are common in children. Most children have a straightforward presentation in the Emergency Department (ED) and are treated with oral antibiotics (by mouth). However, many children with a UTI have some complicated features (eg. fever, vomiting, dehydration, kidney abnormalities). For these children, the length of intravenous (IV) antibiotics treatment (drip antibiotics) is unclear. Although routine care means a total of 7 days of antibiotics, some children receive only 1 dose of IV antibiotics with the remaining 6 days by mouth and some children receive 3 doses of IV antibiotics and the remaining 4 days by mouth. Therefore this study aims to investigate the appropriate duration of antibiotics through a randomised control trial. We are aiming to see if 1 dose of IV antibiotics is as good as 3 doses of IV antibiotics for children with UTI with complicated features.

2. **Who is running the project?**

This project is being led by Dr Laila Ibrahim a paediatrician at the Royal Children’s Hospital (RCH) and a clinical researcher at the Murdoch Children’s Research Institute (MCRI). It will be conducted at the RCH, and other hospitals across Australia and New Zealand.

This project is funded by the National Health and Medical Research Council and The Royal Children’s Hospital Foundation.

3. **Why is my child being asked to take part?**

You and your child are being asked to take part because the nurse or doctor who has seen your child in the ED has diagnosed your child with a UTI with some complicated features (eg. fever, vomiting, dehydration, kidney abnormalities) and your child’s nurse or doctor in the ED determined that your child needs IV antibiotics (drip antibiotics).

4. **What does my child need to do in this project?**

Your child will be in the project for approximately 1 month.

**Randomisation**

We will put your child into one of two groups:

- Group 1. In this group your child will be given 1 dose of IV antibiotics *plus* 6 days of oral antibiotics.
- Group 2. In this group your child will be given 3 doses of IV antibiotics *plus* 4 days of oral antibiotics.

This will be done by chance, similar to tossing a coin, so your child will have an equal chance of being in either group. Neither you or your doctor can choose which group you are put in.

**Assessments**

Participation in the trial will involve the following:

- We will ask you to monitor your child’s temperature 3 times a day (morning, afternoon, night) for 3 days and record whether your child had a fever, shakes/chills or vomiting. Then 3 days after the first dose of IV antibiotics, we will contact you by phone or telehealth to ask you whether your child still has fever, shakes/chills or vomiting. Thermometers will be provided to monitor your child’s temperature and training for using the thermometer will be provided.
- We will collect data from your child’s medical and hospital records such as if your child has any underlying illness, and your child’s use of hospital care such as how long your child remained in hospital or ED and the associated cost. This may also include contacting your GP or other clinical services to obtain further information about the urine sample.
- We will ask you to complete participant and carer well-being short questionnaire three times –as soon as your child is enrolled in the study, 3 days after the first dose of IV antibiotics and 2 weeks after the first dose of antibiotics. The questionnaire will ask about how your child and you are generally feeling.
- We will ask you to also record any extra costs you had to spend during the hospital admission such as parking, medication, child-minding and food. This is to find out how much a hospital admission costs to families.
- We will contact you at 2 weeks and 1 month after the first dose of IV antibiotics by phone or telehealth to ask if your child has had another UTI or if your child has had another hospital admission.
- **There are no extra tests or blood tests in this study.**

|  | **Day 1** | **Day 2** | **Day 3** | **72 hours after**  **first IV dose** | **Day 14** | **1 month** |
| --- | --- | --- | --- | --- | --- | --- |
| **Monitor child’s temperature** | ✔ | ✔ | ✔ |  |  |  |
| **Well-being**  **questionnaire** | ✔ |  |  | ✔ | ✔ |  |
| **Record extra costs** |  |  |  |  | ✔ |  |
| **Phone/telehealth** | ✔ |  |  | ✔ | ✔ | ✔ |

**Optional Consent**

If your child take part in this project, we will ask you to think about consenting to a couple of extra things. These things are optional. The first one is letting us use your child’s information for future projects about children with complicated UTI. The second one is letting us contact you about new research projects. You can say no. If you say no, you can still take part in this project.

a. First optional consent: Use of your child’s data in future research

We will collect information about your child’s hospital visit and antibiotic use. We could answer some of these questions in the future by using the information from this project in a different way. We would like you to consider letting us use your child’s information in future research projects. We will only use the information in research projects that have been approved by the Human Research Ethics Committee. If you agree to this we will use your child’s information without contacting you.

b. Second optional consent: Future contact about new research projects

We would like you to consider letting us send your child’s information about new research projects that may be suitable for your child. The information we send will give you the full details about the project. It is your choice whether you agree to let your child take part in any future project or not. You are not obliged to take part in any future research you are sent information about.

5. **Can my child stop taking part in the project?**

Your child can stop taking part in the project at any time. You just need to tell us so. You do not need to tell us the reason why. If you leave the project we will use any information already collected unless you tell us not to.

6. **What are the possible benefits for my child and other people in the future?**

Your child’s participation in this project will not benefit them personally because they will receive routine care similar to if they were not in the project – treatment with antibiotics (antibiotics for a total of 7 days) and clinical monitoring until the UTI has resolved (discharge).

However, we hope that the project will benefit other children who have complicated UTI in the future, by showing if 1 dose of IV antibiotics is as good as 3 doses of IV antibiotics. This is an important outcome that may benefit children and their families by reducing the duration of IV antibiotics and number of days in hospital.

7. **What are the possible risks, side-effects, discomforts and/or inconveniences?**

Medical treatments often have side effects, as your child has a UTI and requires antibiotics the risk of these side effects are the same whether your child is enrolled in this research study or not. As your child requires IV (drip)antibiotics there is a risk of slight discomfort while the IV line is being inserted or in place. In addition to this if the intravenous line is not accessible then your doctor may decide to give these required antibiotics via an injection into the muscle. This intramuscular injection may be slightly painful for your child. The type and number of antibiotics will depend on what your child’s treating doctor thinks is most appropriate given your child’s medical history, other medications or allergies. These above possible risks or side effects may occur regardless of whether or not your child is involved in the study as it is part of routine clinical practice. Some antibiotics may have no side effects, some or all of the side effects listed below. These side effects may be mild, moderate or severe. If your child has any of these side effects or you are worried about them, please talk to us. We will also be looking out for side effects.

Common side effects (affecting approximately 1% of the population):

- Gastrointestinal (i.e. diarrhoea, nausea, abdominal pain)
- Headache
- Dizziness
- Skin rash

There may be side effects that we do not expect or know about as with any antibiotics. Please tell us immediately if your child experiences any new or unusual symptoms. Many side effects go away shortly after treatment ends. However, sometimes they can be serious, long lasting or permanent. If a severe side effect or reaction occurs, we may need to stop your child’s treatment. If you or your child would like any further information about each antibiotic then please ask and we will happily provide the Product Information for each antibiotic.

The well-being questionnaire may be an inconvenience, but this can be completed online and should not take more than 5 minutes to complete.

8. **What will be done to make sure my child’s information is confidential?**

In this project we will collect and use your child’s personal and health information for research purposes. Any information we collect that can identify you child or your family will be treated as confidential and used only in this project unless otherwise specified. We can disclose the information only with your permission, except as required by law.

To collect information correctly and to be able to contact you for follow up, your child’s hospital number and contact details will be entered into the project database during data collection. These will be removed from the database when data collection finishes. The information stored will be re-identifiable. This means that in the project database we will remove your child’s hospital number and give the information a unique ID number. Only the research team can match your child’s hospital number to your ID number, if it is necessary to do so. Research data will be stored securely in a password protected online database (REDCap). REDCap is hosted by the Murdoch Children’s Research Institute, a partner with The Royal Children’s Hospital at the Melbourne Children’s Campus – the researchers work across both places. A separate code-break document that will store identifiable patient information will be kept on a password-protected research computer.

The following people may access information collected as part of this research project:

•	The research team involved with this project at [Insert site here]

•	The research team involved with this project at The Royal Children’s Hospital

•	The Royal Children’s Hospital Human Research Ethics Committee

We are required to keep information collected as part of a research project for a certain length of time. Because the participants in this project are under 18 years old, we must keep information until the youngest participant turns 25 years old. The research information may be destroyed or kept indefinitely in secure storage after this time.

You have the right to access and correct the information we collect and store about your child. This is in accordance with relevant Australian and/or Victorian privacy and other relevant laws. Please contact us if you would like to access this information.

At the end of the study, we may present the results at conferences. We may also publish the results in medical journals. This will be done in such a way that your child cannot be identified.

9. **Will I be informed of the results when the research project is finished?**

We will send you a summary of the overall project results. The summary will be of the whole group of research study participants, not your child’s individual results.

**10.	Further information and who to contact**

The person you may need to contact will depend on the nature of your query. If you want any further information concerning this project or if your child has any medical problems which may be related to your involvement in the project (for example, any side effects) you can contact any of the following people:

**Clinical contact person**

| Name | Dr Laila Ibrahim |
| --- | --- |
| Position | Principal Investigator |
| Telephone | 03 93455522 through RCH switch |
| Email | Laila.ibrahim@rch.org.au |

**Local Site Clinical Contact Person**

| Name | *[Name]* |
| --- | --- |
| Position | *[Position]* |
| Telephone | *[Phone number]* |
| Email | *[Email address]* |

For matters relating to research at the site at which you are participating, the details of the local site complaints person are below. You can contact the Director of Research Ethics & Governance at The Royal Children’s Hospital Melbourne if you:

- have any concerns or complaints about the project
- are worried about your child’s rights as a research participant
- would like to speak to someone independent of the project.

**Director of Research Ethics & Governance contact person**

| Position | The Director, Research Ethics and Governance, The Royal Children’s Hospital |
| --- | --- |
| Telephone | 03 9345 5044 |
| Email | Rch.ethics@rch.org.au |

**Local HREC Office contact ([Insert site here] Site - Research Governance Officer)**

| Name | *[Name]* |
| --- | --- |
| Position | *[Position]* |
| Telephone | *[Phone number]* |
| Email | *[Email address]* |

**CONSENT FORM**

| **HREC Project Number:** | 72065 | | |
| --- | --- | --- | --- |
| **Short Name of Project:** | CHOICE UTI: Comparing early switch to oral antibiotics after single dose IV (drip) antibiotics to 3 doses IV antibiotics for children with complicated urinary tract infections | | |
| **Version Number:** | 5 | **Version Date:** | 24/11/2022 |

- I have read this information statement and I understand its contents.
- I understand what my child has to do to be involved in this project.
- I understand the risks my child could face because of their involvement in this project.
- I voluntarily consent my child to take part in this research project.
- I have had an opportunity to ask questions about the project and I am satisfied with the answers I have received.
- I understand that this project has been approved by The Royal Children’s Hospital Melbourne Human Research Ethics Committee as well as [Insert site here]. I understand that the project is required to be carried out in line with the National Statement on Ethical Conduct in Human Research (2007).
- I understand I will receive a copy of this Information Statement and Consent Form.

**Optional consent**

| ☐ I do | ☐ I do not | consent for my child’s information from this project to be stored for use in future ethically approved research related to infection and antibiotics |
| --- | --- | --- |
| ☐ I do | ☐ I do not | consent to be contacted about future research projects related to infection and antibiotics |

| Participant’s Name |  |  |  |  |
| --- | --- | --- | --- | --- |

| Name of Parent or Guardian |  | Parent or Guardian’s Signature |  | Date |
| --- | --- | --- | --- | --- |

| Name of Witness to Participant’s Signature |  | Witness Signature |  | Date |
| --- | --- | --- | --- | --- |

**Declaration by researcher:** I have explained the project to the parent/guardian who has signed above. I believe that they understand the purpose, extent and possible risks of their child’s involvement in this project.

| Research Team Member Name |  | Research Team Member Signature |  | Date |
| --- | --- | --- | --- | --- |

Note: All parties signing the consent form must date their own signature.
