# Supplementary material for: CHOICE-UTI: protocol for a multicentre randomised non-inferiority trial comparing single versus three daily doses of intravenous antibiotics prior to oral therapy for complicated urinary tract infection in children
Source: BMJ Open. 2026 Nov 11;16(11):e119766. doi: 10.1136/bmjopen-2026-119766 (PMC13756224; doi:10.1136/bmjopen-2026-119766)
Supplement: Supplementary file 3 [file bmjopen-16-11-s002.docx]

protocol

**CHOICE UTI Trial**

Clinical efficacy of single dose (daily) IV antibiotics followed by 2 days oral antibiotics compared to 3 doses (daily) IV antibiotics for children with complicated urinary tract infections: a multicentre randomised trial

Version: 10

Date: 7/8/26

Project Reference Number: 72065

Short title: Single dose IV antibiotics for complicated UTI in children

Investigators:

Dr Laila Ibrahim, Royal Children's Hospital Melbourne

A/Prof Penelope Bryant, Royal Children's Hospital Melbourne

Prof Franz Babl, Royal Children's Hospital Melbourne

Dr Sarah McNab, Royal Children's Hospital Melbourne

A/Prof Sandy Hopper, Royal Children's Hospital Melbourne

Prof Andrew Davidson, Royal Children's Hospital Melbourne

Dr Barry Scanlan, University of Melbourne

Dr Cathy Quinlan, Royal Children's Hospital Melbourne

Ms Francesca Orsini, Murdoch Children’s Research /institute

Ms Amanda Williams, Royal Children's Hospital Melbourne

Prof Meredith Borland, Perth Children's Hospital

Dr Ariel Mace, Perth Children's Hospital

Prof Stuart Dalziel, Starship Children's Hospital (Auckland)

Prof Cameron Grant, Starship Children's Hospital (Auckland)

Prof Simon Craig, Monash Health

Dr Peter Gowdie, Monash Health

Dr Amit Kochar, Women's and Children's Hospital (Adelaide)

Dr Scott Sypek, Women's and Children's Hospital (Adelaide)

Dr Li Huang, University of Melbourne

Prof Kim Dalziel, University of Melbourne

Dr Jeremy Furyk, University Hospital Geelong

Dr Charmaine Gray, Flinders Medical Centre (Adelaide)

Sites:

Royal Children's Hospital, VIC

Murdoch Children’s Research Institute, VIC

University of Melbourne, VIC

Monash Children’s Hospital, VIC

Perth Children's Hospital, WA

Starship Children's Hospital, Auckland NZ

Women's and Children's Hospital, SA

University Hospital Geelong, VIC

Flinders Medical Centre, SA

**Document history:**

| **Version Number and Date** | **Summary of changes** |
| --- | --- |
| **1.0 Jan 26, 2021** | Initial Version |
| **2.0 Feb 12, 2021** | Changed title, re-label arms, clarification of intervention |
| **3.0 24 Mar, 2021** | Added justification for non-inferiority margin, definition of Intention-to-treat consistent across protocol |
| **4.0 25 Nov, 2021** | Re-wording of intervention for clarity, additional associate investigators |
| **6.0, 10 Jan 2023** | Simplify eligibility criteria, timing and verbal consent for randomisation amended |
| **7.0, 6 Jun 2024** | Clarify definitions, eligibility and exclusion criteria: fever at presentation does not have to be within 8 hours of presentation but is associated with current ED presentation, urine sample collection can be soon after antibiotic treatment, Include definition addition of confirmed UTI - ultrasonographic confirmation of pyelonephritis. Clarify exclusion criteria: recurrent UTI within 2 weeks, clinician only intends on giving 1 dose, unable to obtain consent. |
| **8.0 10 Jun 2025** | Additional site added and clarified the modified intention to treat population |
| **9.0 8 Jan 2026** | Additional site added |
| **10.0 7 Aug 2026** | Amended UTI case definition to include presumed UTI (treating clinician diagnosis and clinical management as UTI, per discharge/follow-up documentation, in addition to confirmed UTI by urine culture or radiological evidence); revised planned sample size to 396 participants (198 per arm) based on updated protocol deviation rate. |

**Statement of Compliance**

This trial will be conducted in compliance with all stipulation of this protocol, the conditions of the ethics committee approval, the NHMRC National Statement on ethical Conduct in Human Research (2007 and all updates), the Integrated Addendum to ICH E6 (R1): Guideline for Good Clinical Practice E6 (R2), dated 9 November 2016 annotated with TGA comments and the NHMRC guidance Safety monitoring and reporting in clinical trials involving therapeutic goods (EH59, 2016).

Table of Contents

[PROTOCOL SYNOPSIS	8](#_Toc119912581)

[GLOSSARY OF ABBREVIATIONS & TERMS	11](#_Toc119912582)

[1.	ADMINISTRATIVE INFORMATION	11](#_Toc119912583)

[1.1.	Trial registration	11](#_Toc119912584)

[1.2.	Sponsor	11](#_Toc119912585)

[1.3.	Funding	11](#_Toc119912586)

[1.4.	Projected timelines	12](#_Toc119912587)

[1.5.	Stakeholder involvement	12](#_Toc119912588)

[2.	INTRODUCTION AND BACKGROUND	12](#_Toc119912589)

[2.1.	Trial rationale and aim	12](#_Toc119912590)

[2.2.	Background	12](#_Toc119912591)

[2.3.	Risk/benefit assessment	15](#_Toc119912592)

[3.	TRIAL OBJECTIVES AND OUTCOMES	16](#_Toc119912593)

[3.1.	Objectives	16](#_Toc119912594)

[3.1.1.	Primary objective	16](#_Toc119912595)

[3.1.1.	Secondary objectives	16](#_Toc119912596)

[3.2.	Outcomes	17](#_Toc119912597)

[4.	STUDY DESIGN	20](#_Toc119912598)

[4.1.	Overall design	20](#_Toc119912599)

[4.2.	Justification for dose	20](#_Toc119912600)

[4.3.	Trial population	22](#_Toc119912601)

[4.4.	Number of participants	23](#_Toc119912602)

[4.5.	Eligibility criteria	23](#_Toc119912603)

[4.5.1.	Inclusion criteria	23](#_Toc119912604)

[4.5.2.	Exclusion criteria	24](#_Toc119912605)

[4.6.	Screen failures	25](#_Toc119912606)

[4.7.	Recruitment and identification of potential participants	25](#_Toc119912607)

[4.8.	Consent	25](#_Toc119912608)

[5.	INTERVENTION	26](#_Toc119912609)

[5.1.	Trial Intervention(s)	28](#_Toc119912610)

[5.1.1.	Description of antibiotics	28](#_Toc119912611)

[5.1.2.	Measurement of participant compliance	30](#_Toc119912612)

[5.1.3.	Excluded medications and concomitant therapy	31](#_Toc119912613)

[6.	RANDOMISATION	31](#_Toc119912614)

[6.1.	Schedule of assessments	31](#_Toc119912615)

[6.2.	Notes on specific trial visits	33](#_Toc119912616)

[6.2.1.	Screening	33](#_Toc119912617)

[6.2.2.	Final trial visit	33](#_Toc119912618)

[6.2.3.	Unscheduled visit	33](#_Toc119912619)

[6.3.	Participant withdrawal, replacement and losses to follow-up	34](#_Toc119912620)

[6.3.1.	Withdrawal of consent	34](#_Toc119912621)

[6.3.2.	Losses to follow-up	34](#_Toc119912622)

[6.3.3.	Replacements	34](#_Toc119912623)

[6.3.4.	Trial Closure	34](#_Toc119912624)

[6.3.5.	Continuation of therapy	35](#_Toc119912625)

[7.	SAFETY EVENTS AND RISKS	35](#_Toc119912626)

[7.1.	Adverse events – definitions	35](#_Toc119912627)

[7.2.	Documentation of adverse events	36](#_Toc119912628)

[7.3.	Assessing the seriousness of a participant’s adverse event	36](#_Toc119912629)

[7.4.	Reporting of safety events	37](#_Toc119912630)

[8.	DATA MANAGEMENT	38](#_Toc119912631)

[8.1.	Data Collection, processing, and storage	38](#_Toc119912632)

[8.1.1.	Source Data	38](#_Toc119912633)

[8.1.2.	Unique participant identifier	39](#_Toc119912634)

[8.1.3.	Data Capture Methods and Storage	39](#_Toc119912635)

[8.1.4.	Record Retention	39](#_Toc119912636)

[9.	STUDY OVERSIGHT	40](#_Toc119912637)

[9.1.	Governance structure	40](#_Toc119912638)

[9.1.1.	Trial Management Group (TMG)	40](#_Toc119912639)

[9.1.2.	Trial Steering Committee (TSC)	40](#_Toc119912640)

[9.1.3.	Safety Monitoring	40](#_Toc119912641)

[9.2.	Quality Control and Quality Assurance	41](#_Toc119912642)

[10.	STATISTICAL METHODS	41](#_Toc119912643)

[10.1.	Sample Size Estimation	41](#_Toc119912644)

[10.2.	Justification of Non-Inferiority Margin	42](#_Toc119912645)

[10.3.	Population to be analysed	45](#_Toc119912646)

[10.4.	Methods of analysis	45](#_Toc119912647)

[11.	ETHICS AND DISSEMINATION	47](#_Toc119912648)

[11.1.	Research Ethics Approval & Local Governance Authorisation	47](#_Toc119912649)

[11.2.	Amendments to the protocol	47](#_Toc119912650)

[11.3.	Protocol Deviations and Serious Breaches	47](#_Toc119912651)

[12.	CONFIDENTIALITY	48](#_Toc119912652)

[13.	PARTICIPANT REIMBURSEMENT	49](#_Toc119912653)

[14.	FINANCIAL DISCLOSURE AND CONFLICTS OF INTEREST	49](#_Toc119912654)

[15.	DISSEMINATION AND TRANSLATION PLAN	49](#_Toc119912655)

[16.	ADDITIONAL CONSIDERATIONS	49](#_Toc119912656)

[17.	REFERENCES	49](#_Toc119912657)

# PROTOCOL SYNOPSIS

| Title | Clinical efficacy of single dose (daily) IV antibiotics followed by 2 days oral antibiotics compared to 3 doses (daily) IV antibiotics for children with complicated urinary tract infections: a multicentre randomised trial |
| --- | --- |
| Study design | An open label, multi-centre, pragmatic, non-inferiority randomised controlled trial (RCT).  It will incorporate a two-arm, non-inferiority design with parallel groups and 1:1 allocation of children presenting to the ED in whom clinicians deem parenteral antibiotics are required for a febrile complicated UTI. |
| Primary objective | The primary objective of this trial is to compare whether **1 dose of a daily parenteral antibiotic followed by 2 days oral** **antibiotics** is as clinically effective (non-inferior) in resolving UTI symptoms at 72 hours after the first IV dose, as **3 doses of a daily parenteral antibiotic** for complicated urinary tract infections presenting to the ED.  **Definition:**  **1 dose:** one dose of a daily dose IV to cover Gram negative bacteria  +/- one dose IV to cover Enterococcus spp. This will be followed by two days oral antibiotics.  **3 doses:** three doses of a daily dose IV to cover Gram negative bacteria +/- 3 days IV antibiotics to cover Enterococcus spp. |
| Secondary objectives | The secondary objectives of this trial are to compare the following outcomes between the **1 dose** and the **3 doses** arms:   - Readmission due to persistent fever, vomiting, rigors, or clinical deterioration (e.g., poor feeding, dehydration) within 14 days of the initial dose of IV antibiotics. - Readmission due to persistent fever, vomiting, rigors, or clinical deterioration (e.g., poor feeding, dehydration) within 1 month of the initial dose of IV antibiotics. - Transfer from HITH/ambulatory care to ward care during admission within 72 hours of the initial dose of IV antibiotics. - Time to resolution of fever/vomiting/rigors within 72 hours of the initial dose of IV antibiotics as reported by parents/guardian. - Improvement as determined by parents/guardian at 72 hours after the first IV dose. - Antiemetic use for 72 hours from the initial IV antibiotic dose. - Duration of IV antibiotics (actually received by patient) from the first dose of IV antibiotics to last dose of IV antibiotics. - Duration of oral antibiotics (actually received by patient) from the first dose of oral antibiotics (after IV antibiotics started) to last dose of IV antibiotics. - Total duration of antibiotics: sum of duration of IV antibiotics and oral antibiotics. - Recurrence of UTI within 14 days from the first dose of IV antibiotics. - Recurrence of UTI within 1 month from the first dose of IV antibiotics. - Complications of UTI from the initial IV antibiotics dose to 14 days after the initial dose. - Adverse events from the initial IV antibiotics dose to 14 days after the initial dose. - Quality of life (QoL) indicators on Day 1 of IV antibiotics (within 24 hours of initial IV antibiotics) and after Day 4 (72 hours up to Day 7). - Parental QoL on Day 14. - Cost-effectiveness. - Follow up microbiological urine culture after commencing IV antibiotics. |
| Outcomes | Primary outcome: Clinical failure defined as *persistence of baseline symptoms* (**fever or vomiting or rigors**) or *development of new symptoms* (**fever or vomiting or rigors**) *attributable to UTI*: at 72 hours from the first dose of IV antibiotics assessed at least 6 hours after last antipyretic. Presence of **fever, vomiting, rigors** reported by parents within 6 hours of assessment will be recorded as present at assessment.  Whether development of new symptoms is attributable to UTI will be judged by an independent blinded clinician.  Secondary outcomes:   - Readmission within 14 days and 1 month. - Transfer from HITH/ambulatory care to ward care - Time to resolution of fever/vomiting/rigors - Antiemetic use for 72 hours - Duration of IV antibiotics - Duration of oral antibiotics - Total duration of antibiotics - Recurrence of UTI within 14 days and 1 month. - Complications - Adverse events - Quality of life (QoL) indicators - Cost-effectiveness - Microbiological urine culture - Renal abnormalities on ultrasound |
| Planned sample size | A total of 396 participants will be enrolled and randomised in the study (approximately 198 per treatment arm). |
| Study population | Patients aged 3 months (corrected age) to 18 years (<19 years) who have presented to the ED febrile complicated UTI (1 of the following complicating features: history of recurrent UTI, urological abnormalities, vomiting, rigors, and tachycardia) |
| Study duration | The study is expected to be completed within 5 years from the start of recruitment. However, delays have meant the commencement of recruitment will vary by site. Once recruitment commences, it is expected to take at least 3 years for each site to complete recruitment. |
| Intervention | Patients who are eligible for the study will be randomised to receive 1 dose of daily IV antibiotics followed by 2 days of oral antibiotics or 3 doses of daily IV antibiotics. All participants will receive a total of 7 days treatment with antibiotics for the complicated urinary tract infection. After the initial three days of treatment, a further four days of oral antibiotics will be continued.  Oral antibiotics will start as soon as able to tolerate, within 12 hours of the last IV dose for both arms. |
| Study methodology | Enrolment and randomisation: During ED assessment, clinicians will identify patients with a suspected febrile complicated UTI requiring treatment with IV antibiotics and screen patients against eligibility criteria. The relevant clinical team at each site will receive standardized, study specific education based on centrally developed study education materials. Participants will be randomised to one of the 2 arms after informed consent is obtained.  Intervention Day 1:  The first dose of the antibiotics will be commenced in the ED. Care of the patient will be as per routine clinical care. Decision for admission to hospital and location of treatment (ambulatory/Hospital-in-the-Home care) will be determined by the treating clinician as per routine clinical care. Parents will be provided with a thermometer and a diary to record a daily assessment of their child (fever, rigors, vomiting).  Intervention Day 4 :  72 hours after the first dose of IV antibiotics, a research nurse will conduct an assessment over phone or telehealth to obtain the primary outcome data: Persistence of baseline symptoms (fever, vomiting, rigors) or development of these symptoms (if not previously present) as documented at baseline. Any ambiguity with regards to symptoms being attributable to UTI will be judged by a blinded clinician external to the research team.  Follow up Day 14:  14 days after the first dose of IV antibiotics, a research nurse will conduct an assessment over phone or telehealth for follow up data including whether GP/ED visit or readmission occurred for UTI and total duration of antibiotics taken by the patient.  Follow up 1 month:  1 month after the first dose of IV antibiotics, a research nurse will conduct an assessment over phone or telehealth for follow up data including whether GP/ED visit or readmission occurred for UTI, total duration of antibiotics taken by the patient. |
| Statistical methods – primary outcome | Statistical analysis will follow both intention-to-treat (ITT) and per-protocol (PP) approaches, which is common for non-inferiority randomised controlled trials.  Primary analysis of the primary outcome: The number and proportion of participants who will be assessed as treatment failures within 72 hours from the first dose of IV antibiotics will be summarised by treatment arm, using absolute and relative frequencies.  The risk difference with 95% confidence interval will be estimated using a generalised linear model to adjust for the strata used during randomisation as a covariate. For the **1 dose** to be non-inferior to **3 doses**, the upper limit of the two-sided 95% CI of the risk difference must be less than 10%. |

# GLOSSARY OF ABBREVIATIONS & TERMS

| **ABBREVIATION** | **TERM** |
| --- | --- |
| AE | Adverse event |
| CRDO | Clinical Research Development Office |
| CPG | Clinical Practice Guideline |
| cUTI | Complicated urinary tract infection |
| ED | Emergency Department |
| GCP | Good clinical practice |
| HITH | Hospital-in-the-home or other home-based care |
| HREC | Human Research Ethics Committee |
| IM | Intramuscular |
| IV | Intravenous |
| MCRI | Murdoch Children’s Research Institute |
| MCTC | Melbourne Clinical Trials Centre |
| PREDICT | Paediatric Research in Emergency Department International Collaborative |
| RCH | Royal Children’s Hospital |
| RCT | Randomised Controlled Trial |
| UTI | Urinary Tract Infection |
| uUTI | Uncomplicated urinary tract infection |
| QoL | Quality of Life |

# 1. ADMINISTRATIVE INFORMATION

# 1.1. Trial registration

Clinicaltrials.gov, ID NCT04876131

# 1.2. Sponsor

Murdoch Children’s Research Institute Clinical Trials Sponsorship Committee

# 1.3. Funding

Name of grant/sponsor:

1) The Royal Children’s Hospital Foundation Research Grant 2016-718
2) National Health and Medical Research Council MRFF Research Grant MRF1200411

Confirmed/sought: Confirmed

# 1.4. Projected timelines

The anticipated duration of the trial is 5 years for recruitment.

# 1.5. Stakeholder involvement

During the planning phase, the trial investigators liaised with stakeholders, both at our local institution and through the Paediatric Research in Emergency Department International Collaborative (PREDICT) network, to design a project of clinical significance with potential for practice change. The project plan was reviewed by the PREDICT network during October-November 2019. The review generated feedback addressing the project’s clinical significance, methodological rigor, and outcomes. The project has also been presented to members of the Melbourne Children’s Trial Centre (MCTC) on several occasions during 2019. Feedback was provided pertaining to clinical significance, methodological rigor, outcomes, and outcome measures.

Through these collaborations, Emergency Department (ED) clinicians and general paediatricians collectively expressed the persistent practice of using intravenous (IV; or intramuscular (IM) when IV access is difficult) antibiotics for urinary tract infection (UTI), despite evidence for the use of oral antibiotics for patients with uncomplicated UTI. These discussions highlighted the lack of studies that have included patients with complicated UTI. Therefore, this study attempts to address a common question raised by healthcare providers regarding optimal management of complicated childhood UTIs, which is relevant to clinicians worldwide and more importantly to patients and their families. Hence, a consumer representative has also been involved in the development of the study, specifically with the outcome measure most important to families, their child ‘feeling better'.

# 2. INTRODUCTION AND BACKGROUND

# 2.1. Trial rationale and aim

Urinary tract infections are highly prevalent in the paediatric population, where 3% of children have at least one urinary tract infection (UTI) in their childhood (1). Most cases are relatively straightforward, requiring oral antibiotics. However, a sub-sample of these children present with co-existing complications such as vomiting, dehydration and urological abnormalities. For these children, there are no clinical guidelines to direct practice, which results in considerable variation of care and prolonged courses of antibiotics leading to antimicrobial resistance.

This pragmatic trial, reflecting real world clinical practice, aims to investigate whether **One dose –** **single dose of IV followed by 2 days oral** antibiotics is as non-inferior to **Three doses** for children with UTI and co-existing complicating factors presenting to the ED.

# 2.2. Background

Three percent of children have at least one UTI in their childhood (1). It is the most common reason in the developed world for children to need antibiotics, accounting for 1.2% of all problems managed in Australian general practice (2), and 12% of preventable hospitalisations across all age groups (3). The most frequent reason for children with UTI requiring hospitalisation is for IV antibiotics and this constitutes a major burden for healthcare systems (4). For those without complicating factors, there is good evidence for treatment with oral antibiotics at home (5-8).

For children who present with co-existing complications such as vomiting, dehydration and urological abnormalities, there is little evidence for route of antibiotic treatment or treatment duration because trials have focused on those with uncomplicated UTI. Children with complicating features (from here on referred to as complicated UTI (cUTI)) have been specifically excluded from trials and therefore from Cochrane reviews of IV and oral duration (9). And yet cUTI accounts for about 70% of all children presenting with UTI to emergency departments (ED), *de novo* or via their GP, representing 7,500/year in Australia and New Zealand, many of whom are treated with IV antibiotics (10, 11). Major guidelines make no clear recommendations, beyond that 2-4 days is ‘usually used’ (10, 11). The appropriate duration of IV antibiotics for cUTI in children is not known and it has never been subjected to a randomised controlled trial (RCT).

**Why has cUTI not been included in previous trials?**

- Difficulties in objectively describing the severity/complexity of patients to define the population: different studies have used different features and numbers of features to describe ‘complicated’;
- Physicians are anxious about undertreating these children in case their condition worsens or risks damage to their kidneys;
- There is a pervasive belief that IV antibiotics are better than oral antibiotics. The way in which IV antibiotics are ‘better’ for UTI is that they act faster in the initial delivery to the site of infection because this avoids gut transit, digestion and absorption. However, the dogma that ‘more days of IV is better’ is strong, and many physicians will continue IV until a child is no longer febrile, a tenet that has no evidence to support it. In fact, patients with UTI and fever alone have been specifically shown not to require IV antibiotics at all (9).
- Previous trials in UTI have had wide variability in the inclusion criteria and outcome assessment rendering comparisons between trials difficult. The available guidance for UTI trial design for adults appears not to be used by paediatricians and do not seem applicable to childhood UTI. A systematic review on febrile UTI clinical trials called for a harmonised design for paediatric UTIs (12).

**Proposed new trial**

We propose to address this lack of evidence with the first RCT to compare whether **One dose -** **single dose of IV followed by 2 days oral** antibiotics is as clinically effective (non-inferior) in resolving UTI symptoms at 72 hours after the first IV dose as **Three doses** for complicated urinary tract infections presenting to the ED, with the same total duration (7 days) completed. This is based on scientific plausibility, data from our foundation studies and recommendations from a systematic review on febrile UTI clinical trials.

**1) Scientific plausibility**

The pharmacokinetics of IV antibiotics mean they reach their maximum concentration in blood more quickly than oral antibiotics (13). The goal is initial rapid antibiotic delivery because delay in treatment initiation is a risk factor for permanent renal scars after the first episode of UTI with fever.

There is, however, no evidence for the need for multiple IV antibiotic doses. A recent systematic review of single dose IV antibiotics included children, but the studies were predominantly for uncomplicated UTI (uUTI), not randomised and the most recent study was 3 decades ago (14). In the two non-randomised studies of cUTI, a single dose was associated with more relapses, but there were no subsequent oral antibiotics, just one dose of IV therapy. We propose to use initial IV to act as a loading dose, then switch to oral antibiotics. Oral antibiotics after an IV dose will keep the antibiotic level above the minimum inhibitory concentration for bacterial killing (15, 16).

**2) Foundation studies**

The proposed new trial has been meticulously planned with stakeholder engagement over the past 4 years in 3 phases. This new trial builds on previous completed research.

***Phase 1*** *(2016-2018)*: *Investigation of the current management of UTI* (17-19).

An observational study of 1,235 children attending The Royal Children’s Hospital (RCH) ED with confirmed or probable UTI (17-19). Of these, 70% had at least one complicating feature although only 27% were treated with initial IV antibiotics.

The majority of children with cUTI were treated with IV (83%) received 2-4 days IV antibiotics (85% in hospital, 15% in our home IV programme). Nineteen percent were admitted to hospital but then switched to oral after 1 dose - equivalent to one day, as UTI is treated with IV gentamicin or IV ceftriaxone which are both administered once daily.

Unexpectedly, we found that, for 20% of children, physicians gave 1 dose IV as a rapid dose before sending the child home from ED on oral antibiotics. This is not recommended in any guidelines and reflects the uncertainty physicians face and the belief by experienced ED physicians that these children do not all need to be in hospital. The clinical features of severity at presentation were not distinguishable between the children receiving 1 IV dose and the children receiving 2-4 days.

The average duration of subsequent oral antibiotics in each cohort was the same, but varied between total antibiotics for 7-14 days. The efficacy and readmission rate (4%) were also the same in both groups. There is, therefore, equipoise between IV antibiotics for 1 dose and multiple days for childhood cUTI – the ideal situation for an RCT.

***Phase 2*** *(2017-2018)*: *Derivation and validation of a method using clinical features of severity and complexity* *to objectively define the term ‘complicated’ for UTI.* (19)

From this large study of 1,235 children, the combination of clinical features that most reliably predicted use of IV antibiotics were the following: vomiting, fever, tachycardia, rigors, urological abnormalities, recurrent UTI (area under curve 0.85, 95% CI 0.79-0.91).

These findings are consistent with the fact that many of these features were used to exclude children from previous studies of uUTI (5-8). The more complicating features patients had, the more likely they were to receive IV. Based on recent data, from the period of June to mid Oct 2022, almost all children who were treated with IV antibiotics were febrile with at least one additional feature. Of 224 children who presented to ED with UTI, 26 (12%) were treated with IV antibiotics; 22 of these were children who were febrile with at least 1 additional feature identified above. Thus, patients with cUTI requiring IV were defined by having a febrile UTI with 1 of the following complicating feature vomiting, tachycardia, rigors, urological abnormalities, recurrent UTI.

**3) Urinary Tract Infection Antibiotic Trial Study Design: A Systematic Review (12)**

Basmaci *et al(12)* recommended a primary outcome of concomitant clinical (clinical cure defined as defervescence) and microbiological evaluation at 48-72 hours after starting treatment. Our proposed primary outcome will be based on Basmaci’s recommendation (will include defervescence at 72 hours) but to account for those who did not have fever at inclusion, other UTI symptoms will be assessed: vomiting and rigors. Measuring presence of fever, vomiting and rigors speaks directly to a child ‘feeling better’. Microbiological sampling/evaluation at 48-72 hours after starting treatment in otherwise clinically recovering children is not practical, not ethical, and irrelevant to clinical practice (not usually done in real practice).

# 2.3. Risk/benefit assessment

Risks:

- One dose - may result in prolonged symptoms or treatment failure

Benefits:

- Reduced number of days of IV antibiotics (reduced amount of IV antibiotics and its associated risks)
- Reduced number of days in hospital (reduced duration of hospital admission and its associated risks)

**What are the consequences of not investigating optimal treatment for cUTI?** *There are important detrimental outcomes:*

- Considerable unwarranted variation in care which leads to worse patient outcomes (17, 20). In an era of evidence-based practice, it is unacceptable to have a common condition treated without evidence.
- Children admitted to hospital who could go home. We have shown extensively that treatment at home results in the same or better clinical outcomes, children are happier, they sleep better, quality of life improves, and their parents are less stressed and financially better off (21-24).
- Children, already among the highest users of antibiotics (25), receiving excessive durations of IV antibiotics. This drives antimicrobial resistance (AMR), a WHO decreed global and increasing healthcare crisis (26), IV access complications and can be associated with chronic ill health (27).
- Substantial wastage of hospital resources, the antithesis of sustainable healthcare. We have previously shown the dominant cost-effectiveness of reducing hospital IV antibiotics (24).

# 3. TRIAL OBJECTIVES AND OUTCOMES

# 3.1. Objectives

# 3.1.1. Primary objective

The primary objective of this trial is to compare whether **One dose -** **single dose of IV *followed by 2 days oral*** antibiotics is as clinically effective (non-inferior) in resolving UTI symptoms at 72 hours after the first IV dose as **Three doses** for complicated urinary tract infections presenting to the ED.

**Definition:**

**One dose**: Single dose IV to cover Gram negative bacteria followed by 2 days oral antibiotics +/- Single dose IV to cover Enterococcus spp

**Three doses:** 3 doses IV to cover Gram negative bacteria

+/- 3 days IV antibiotics to cover Enterococcus spp

# 1.1.1. Secondary objectives

The secondary objectives of this trial are to compare the following outcomes between the **One dose** and the **Three doses** arms:

1. Readmission due to persistent fever, vomiting, rigors, or clinical deterioration (e.g., poor feeding, dehydration) within 14 days of the initial dose of IV antibiotics.
2. Readmission due to persistent fever, vomiting, rigors, or clinical deterioration (e.g., poor feeding, dehydration) within 1 month of the initial dose of IV antibiotics.
3. Transfer from HITH/ambulatory care to ward care during admission within 72 hours of the initial dose of IV antibiotics.
4. Time to resolution of fever/vomiting/rigors within 72 hours of the initial dose of IV antibiotics as reported by parents/guardian.
5. Improvement as determined by parents/guardian at 72 hours after the first IV dose.
6. Antiemetic use for 72 hours from the initial IV antibiotic dose.
7. Antipyretics/analgesia use for 72 hours from the initial IV antibiotic dose.
8. Duration of IV antibiotics (actually received by patient) from the first dose of IV antibiotics to last dose of IV antibiotics.
9. Duration of oral antibiotics (actually received by patient) from the first dose of oral antibiotics (after IV antibiotics started) to last dose of IV antibiotics.
10. Total duration of antibiotics: sum of duration of IV antibiotics and oral antibiotics.
11. Recurrence of UTI within 14 days from the first dose of IV antibiotics.
12. Recurrence of UTI within 1 month from the first dose of IV antibiotics.
13. Complications of UTI from the initial IV antibiotics dose to 14 days after the initial dose.
14. Adverse events from the initial IV antibiotics dose to 14 days after the initial dose. Any GP visits, ED presentations and hospital admission length of stay will be recorded resulting from adverse events.
15. Quality of life (QoL) indicators on Day 1 of IV antibiotics (within 24 hours of initial IV antibiotics) and after Day 4 (72 hours up to Day 7).
16. Cost-effectiveness – hospital administrative data on costs and patient/parents reported costs.
17. Follow up microbiological urine culture after commencing IV antibiotics for those who have remaining UTI symptoms (e.g., fever, abdominal pain, nausea/vomiting, dysuria, frequency, urgency) after completion of 7 days of antibiotic course.
18. Imaging reports – ultrasound or other imaging results will be collected for patients who have them performed.

# 1.2. Outcomes

Primary outcome: Clinical failure at 72 hours from the first dose of IV antibiotics, defined as persistence of baseline symptoms (fever or vomiting or rigors) or development of new symptoms (fever or vomiting or rigors) attributable to UTI. Presence of fever, vomiting, rigors reported by parents within 6 hours of assessment will be recorded as present at assessment.

- Whether development of new symptoms is attributable to UTI will be judged by an independent blinded clinician.
- Vomiting - will be judged as present if different to what is expected of the child’s usual condition (e.g., not due to known gastro-oesophageal reflux or cyclical vomiting) and causing parental concern (i.e., not a single vomit). If unclear, will be referred to an independent blinded clinician.
- Fever - measured equal to or more than 38 degrees Celsius using tympanic thermometer.

**Secondary outcomes:**

1. Readmission due to persistent fever, vomiting, rigors, or clinical deterioration (e.g., poor feeding, dehydration) within 14 days of the initial dose of IV antibiotics.

- Admission is defined as patients who are deemed to need hospital funded care/intervention from a hospital-based nurse/doctor (including to HITH/ambulatory care).
- Discharge is defined as when patients admitted to hospital are deemed to no longer require any hospital funded care/intervention from a hospital-based nurse/doctor (including from HITH/ambulatory care).
- Day 14 follow up will be made as close to 14 days after from the initial IV dose as possible but within reason i.e., if Day 14 falls on Saturday, the participant will be contacted on Monday. Date and time of Day 14 follow up will be documented.

2. Readmission due to persistent fever, vomiting, rigors, or clinical deterioration (e.g., poor feeding, dehydration) within 1 month of the initial dose of IV antibiotics.

- 1 month follow up will be made as close to 1 month after from the initial IV dose as possible but within reason i.e., if 1 month falls on Saturday, the participant will be contacted on Monday. Date and time of 1 month follow up will be documented.

3. Transfer from HITH/ambulatory care to ward care during admission within 72 hours of the initial dose of IV antibiotics.
4. Time to resolution of fever/vomiting/rigors within 72 hours of the initial dose of IV antibiotics as reported by parents/guardian.

- Daily parental assessment over 72 hours: In both arms, parents will be asked to prospectively record presence of fever, vomiting, and rigors in a diary which will be provided at enrolment. Hospital grade thermometers will be provided for families. Parents will be shown how to use thermometers at enrolment. Recordings will be done 3 times a day (morning, afternoon, night) without pre-determined times. (Appendix 2).

5. Improvement as determined by parents/guardian at 72 hours after the first IV dose.

- Parent report of ‘generally improved/better’ at 72 hours of the initial IV antibiotic dose.

6. Antiemetic use for 72 hours from the initial IV antibiotic dose.

- Administration of antiemetics will be recorded as ‘yes’ if at least one antiemetic was administered to the patient after the first dose of IV antibiotic, irrespective of dose/frequency.
- Daily administration of antiemetics will be recorded as ‘yes’ if at least one antiemetic was administered to the patient in a 24-hour period starting after the first dose of IV antibiotic, irrespective of dose/frequency.
- Antiemetic use will be recorded from the time of the first dose of IV antibiotic to 72 hours from initial antibiotic dose.

7. Antipyretics/analgesia use for 72 hours from the initial IV antibiotic dose.
   - Administration of antipyretics/analgesia will be recorded as ‘yes’ if at least one antipyretics/analgesia was administered to the patient after the first dose of IV antibiotic, irrespective of dose/frequency.
   - Daily administration of antipyretics/analgesia will be recorded as ‘yes’ if at least one antiemetic was administered to the patient in a 24-hour period starting after the first dose of IV antibiotic, irrespective of dose/frequency.
   - Antipyretics/analgesia use will be recorded from the time of the first dose of IV antibiotic to 72 hours from initial antibiotic dose.
8. Duration of IV antibiotics (actually received by patient) from the first dose of IV antibiotics to last dose of IV antibiotics.
9. Duration of oral antibiotics (actually received by patient) from the first dose of oral antibiotics (after IV antibiotics started) to last dose of oral antibiotics.

- Time in days and proportion of days (1 decimal point) between first dose of oral antibiotics to last dose of oral antibiotics for the same episode of urinary tract infection.

10. Total duration of antibiotics: sum of duration of IV antibiotics and oral antibiotics.
11. Recurrence of UTI within 14 days from the first dose of IV antibiotics.

- Reported by parents - In the last 14 days since the first dose of IV antibiotics, has the patient had recurrence of UTI.

12. Recurrence of UTI within 1 month from the first dose of IV antibiotics.

- Reported by parents - In the last month since the first dose of IV antibiotics, has the patient had recurrence of UTI.

13. Complications of UTI from the initial IV antibiotics dose to 14 days after the initial dose.

- Abscess, sepsis, meningitis from the initial IV antibiotics dose to 14 days after the initial dose.

14. Adverse events from the initial IV antibiotics dose to 14 days after the initial dose.
    - Adverse events (e.g., rash, diarrhoea, medication errors) and clinically determined allergic reactions

- Any GP visits, ED presentations and hospital admission length of stay will be recorded resulting from.

15. Quality of life (QoL) indicators on Day 1 of IV antibiotics (within 24 hours of initial IV antibiotics) and after Day 4 (72 hours up to Day 7).

- The CHU9D score of children with UTI across 9 domains – worried, sad, pain, tired, annoyed, school/homework, sleep, daily routine, and activities on Day 1 of IV antibiotics (within 24 hours of initial IV antibiotics), Day 4 (72 hours up to Day 7) and Day 14. Parental QoL score (EQ-5D) will be obtained at the same timepoints.

16. Cost-effectiveness – hospital administrative data on costs and patient/parents reported costs.
17. Follow up microbiological urine culture after commencing IV antibiotics for those who have remaining UTI symptoms (e.g., fever, abdominal pain, nausea/vomiting, dysuria, frequency, urgency) after completion of 7 days of antibiotic course.
18. Imaging reports – ultrasound or other imaging results will be collected for patients who have them performed.

Further detail of timepoints for assessment are outlined in Table 3 (Section 7).

2. STUDY DESIGN

# 2.1. Overall design

This study is an open label, multi-centre, non-inferiority randomised controlled trial (RCT).

It will incorporate a two-arm, non-inferiority design with parallel arms and 1:1 allocation of children presenting to the ED with a febrile complicated UTI treated with parenteral antibiotics (Figure 1).

**Figure 1. Study design diagram**

UTI = urinary tract infection; m = months; y = years; Antibiotics used in the trial will be as per local antibiotics guidelines and urine microscopy/culture results. Dosing and frequency as per standard local guidelines. Potential antibiotics used listed below in 5.1.1 Description Of Antibiotics. IV refers to IV or IM if IV cannot be sited.

# 2.2. Justification for dose

The RCH Clinical Practice Guidelines (CPG) for the treatment of UTI, as well as the following site guidelines: Monash Children’s Hospital, Perth Children’s Hospital, Starship Children’s Hospital (New Zealand) recommend a 7-10 day course of antibiotics for children with pyelonephritis, with initial IV antibiotics for children who are seriously unwell or infants under 3 months (corrected age) of age (28). Some recommend IV antibiotics for 2-4 days or a switch to oral antibiotics once clinical improvement is observed (29).

A guideline for the practice of IV to oral switch for common infections recommended a total of 10 days for pyelonephritis with a caveat that in a child who rapidly improves 7 days may be sufficient (30). This guidance was based on several studies and included a Cochrane review of acute pyelonephritis in children treated with 10–14 days of antibiotics, that found no difference in duration of fever or renal damage between all intravenous antibiotics, 3 days of intravenous followed by oral antibiotics, or all oral administration (8, 9). The question of the optimal total duration of antibiotics in children with febrile UTI (7 versus 10 days) is currently the subject of an ongoing RCT reflecting the equipoise in this matter (31).

To gauge stakeholder opinion on total duration, we surveyed general paediatricians at RCH (where the guideline for lower tract UTI recommends 3-7 days and upper tract UTI 7-10 days) about how they usually manage paediatric patients with complicated UTI. From 21 respondents, 15 (71%) would administer a 7-day total course of IV antibiotics plus oral antibiotics, 6 respondents a 5-day course and 1 respondent a 10-day course.

There is a lack of clear guidance and no evidence pertaining to treatment for children with complicating features of UTI. We know that most children rapidly improve evidenced by a median length of hospital stay of 2.2 days (unpublished baseline data), therefore we will test a 7-day course of combined IV and oral antibiotics with IV administered for 1 dose (plus 6 days oral) versus 3 doses (plus 4 days oral). All parents/guardians will be advised after their child completes a 7-day course, if the participant is not completely well, they should have a medical review with their general practitioner or Emergency (whichever appropriate) as per routine advice (See Appendix letter to GP/Emergency). At Day 14 follow up, the total duration prescribed will be documented and participants will be asked total duration of oral antibiotics actually taken.

The route and choice of antibiotic (IV and oral) will align with recommendations as per the local guidelines.

Table 2. Antibiotic regimen as per local guidelines*

| Institution | IV antibiotics | Oral antibiotics | Total duration |
| --- | --- | --- | --- |
| Paediatric Improvement Collaborative (RCH, Monash, Safer Care Victoria, Queensland, NSW) | **1 dose**: single dose IV gentamicin +/- single dose IV benzylpenicillin  **3 doses**: 3 doses^#^ IV gentamicin +/- penicillin (length of penicillin as per organism culture, if indicated) | cephalexin  trimethoprim  co-trimoxazole  amoxicillin | 7 days |
| Starship Children’s Hospital, NZ | **1 dose**: single dose IV gentamicin OR single dose IV ceftriaxone  **3 doses**: 3 doses IV gentamicin OR 3 doses IV ceftriaxone | cephalexin  nitrofurantoin  co-trimoxazole  amoxicilin/clavulanic acid  cefuroxime | 7 days |
| Perth | **1 dose**: single dose IV gentamicin AND single dose IV amoxicillin OR single dose IV ceftriaxone only  **3 doses:** 3 doses IV gentamicin +/- IV amoxicillin (length of amoxicillin as per organism culture, if indicated) OR 3 doses IV ceftriaxone only | cephalexin  co-trimoxazole  amoxicilin/clavulanic acid | 7 days |
| Women’s and Children’s Adelaide | **1 dose**: single dose IV gentamicin+/- single dose IV amoxicillin  **3 doses**: 3 doses IV gentamicin+/- 3 days IV amoxicillin  (length of penicillin as per organism culture, if indicated) | cephalexin  co-trimoxazole  amoxicilin/clavulanic acid | 7 days |

*Please note all preparation and administration of these antibiotics will be as per local hospital guidelines. These listed regimens are what is anticipated in most participants. However, amikacin or ceftriaxone may be used in some clinical situations where the regimen described is unsuitable and will not be excluded from the trial. ^#^1 day of a once-daily drug requires 1 dose, 3 days of a once-daily drug requires 3 doses. For multiple doses per day drug, 3 days constitute antibiotic coverage beyond 48 hours. If enterococcus identified, need to give minimum 9 doses of penicillin i.e., 9^th^ IV dose at 48 hours.

# 2.3. Trial population

Patients aged 3 months (corrected age) to 18 years (<19 years) who have presented to the ED with a febrile complicated UTI treated with IV antibiotics (fever plus at least 1 of the following complicating feature vomiting, tachycardia, rigors, urological abnormalities, recurrent UTI)

# 2.4. Number of participants

The study will recruit for 396 children with a 1:1 allocation between the two treatment arms (i.e., 198 participants in each treatment arm).

# 2.5. Eligibility criteria

Patients will be randomised if they meet all of the inclusion criteria and none of the exclusion criteria.

# 2.5.1. Inclusion criteria

Table 3. Criteria for inclusion

|  | **Inclusion criteria** | **Definitions** |
| --- | --- | --- |
| Age | 3 months (corrected age) to 18 years (prior to 19^th^ birthday) |  |
| Febrile complicated UTI | Fever | **Fever:** reported fever at home or measured fever of ≥38 degrees Celsius associated with the illness that triggered current ED presentation (eg fever may have been 18 hours prior to presentation but none since then because patient has been on maximal antipyretics – paracetamol or ibuprofen) . |
| Complicated features | Any of the following:  1. Vomiting   2. Rigors 3. History of recurrent UTI 4. Urological abnormalities 5. Tachycardia | **Vomiting**: reported vomiting different to usual baseline. Vomiting - will be judged as present if different to what is expected of the child’s usual condition (e.g., not due to known gastro-oesophageal reflux or cyclical vomiting) and causing parental concern. If unclear, will be referred to an independent blinded clinician.    **Rigors:** sudden shaking or shivering reported by parents/guardian/child, not due to any other known cause.  **Recurrent UTI**: any upper or lower tract UTI treated with a course of antibiotics. This may be reported by the parents/guardian in ED and clinicians may not have access to previous records.  **Urological abnormalities**: Any functional or anatomical abnormality of the urogenital tract, including but not limited to renal ectopia, vesicoureteral reflux, renal dysplasia, presence of a stent, duplicated collection systems, and hydronephrosis.  **Tachycardia**: measured increased heart rate outside of the age acceptable ranges, as per local vital chart e.g., orange zone as per Victorian Children’s Tool for Observation and Response (ViCTOR). |
| Urine sample available |  | Urine culture must have been collected prior or within an hour of commencement of antibiotic treatment, either at the GP or ED – in order to assess urine culture as per below. |
| Urine criteria  (from recommend-ations by Basmaci et al)(12) | Abnormal urinary dipstick leucocyte esterase >1+ or nitrite positive  OR  ≥5 WBCs per high-power field in centrifuged urine  OR  ≥ 10 WBCs per mm^3^ in uncentrifuged urine and bacteriuria with any bacteria per high-power field | In ED, only urine dipstick or urinalysis will be available. Once urine culture is available, to be included in the efficacy analysis, culture results must meet the following criteria:   - Positive urine culture result with no more than 2 species of microorganisms - AND - Spontaneously voided urine with ≥10^5^ microorganisms per mL of urine or Suprapubic aspirate or urinary catheter with ≥10^4^ microorganisms per mL of urine - In the absence of a positive urine culture, ultrasonographic findings supporting pyelonephritis (per reporting radiologist) will be accepted as evidence of a urinary tract infection In the absence of a positive urine culture or supportive ultrasonographic findings, a case will also be accepted as presumed UTI where the treating clinician has provided a diagnosis of UTI and treated the patient accordingly. |
| ED Clinician assessment | Determines the child requires treatment with IV antibiotics | Systemically unwell, failed oral antibiotics, unable to tolerate oral antibiotics (e.g., vomiting) |

# 2.5.2. Exclusion criteria

Patient:

- Sepsis (requiring inotropic support or more than 20 ml/kg of fluid bolus in ED).
- Known allergy to all once daily study drug options (gentamicin or ceftriaxone or amikacin).
- If the patient has another co-existing condition which requires (based on established evidence-based guidelines) more than 1 dose of IV antibiotics e.g., meningitis.
- Known chronic renal failure or renal transplant patients
- Unrepaired posterior urethral valves.
- Indwelling stent and fever.
- Patients with clinically suspected renal abscess e.g., extreme renal tenderness, out of keeping with pyelonephritis (clinically determined).
- Previously enrolled participants in the CHOICE UTI trial.
- No available oral antibiotic option for this UTI: urine culture result already available and multi-resistant organism with susceptibility only to IV antibiotics or known intolerance to oral antibiotics (previous UTI with multi-resistant organism not an exclusion)
- Previous IV antibiotics for same UTI episode eg interhospital transfer whereby significant time has passed since first dose IV
- Clinician does not intend on prescribing a course of IV antibiotics but plans on only giving a single dose from the outset
- Recurrence of urinary tract infection within 2 weeks
- Unable to obtain consent

# 2.6. Screen failures

Screen failures are defined as participants who could consent to participate in the trial but who are found, during the screening procedures, to be ineligible to continue in the trial. They therefore do not receive the intervention / are not randomised.

Minimal data describing the participant and outcomes will be collected for screen failures. This will include demographic characteristics (age, gender), the reason for ineligibility and outcome measures such as duration of treatment and clinical outcomes.

# 2.7. Recruitment and identification of potential participants

Prior to the trial commencing, ED and ward clinicians (senior doctors, junior doctors or nurse practitioners) will have education sessions to inform them about the study. These education sessions will be integrated into pre-existing staff education sessions. Names of those already attended will be obtained and listed in the delegation log for enrolment and consent. Participation is voluntary.

During assessment, clinicians will identify patients with a suspected UTI presenting to ED. At this stage, the ED or ward clinician will screen patients against eligibility criteria (complicated UTI, meeting inclusion criteria) and may decide to enrol the patient. If the clinician is uncertain of eligibility, they will be encouraged to make contact with a member of the research team.

The parents/guardians of patients (and where applicable the patient) in the complicated UTI group who meet the inclusion criteria will be invited by the researcher or treating clinician to provide their consent to participate in the study. Consent will be for randomisation, data collection and follow up that is not part of routine practice. Consent and randomisation should not delay antibiotic treatment.

# 2.8. Consent

A member of the research team will discuss the trial with relevant family members (parent/legal guardian and where appropriate the child/adolescent participant) and provide the Parent/Guardian or Participant Information and Consent Form, in paper or electronic format. This document will describe the purpose of the trial, the procedures to be followed, and the risks and benefits of participation. During this time the parents/patients will have the opportunity to ask questions about the trial. The paper format will be the same as the electronic format. Due to the evolving COVID-19 pandemic and restrictions on staff and patient movement around hospitals paper or electronic consent from parents and/or patients can be used. The parent or guardian will be able to complete the form on a mobile device, using an electronic signature. The electronic consent form will be stored in the study database (via REDCap).

If the ED is extremely busy, written or electronic consent can be delayed but verbal consent will be obtained and documented in the medical notes prior to randomisation. (See attached recruitment script) If the patient is deemed as having the maturity and understanding sufficient enough to grasp the nature and consequences of the project they may consent to participate. Consent will be documented in the participant’s record. Basic demographic and clinical data (outcome measures available from a patient’s clinical file) will also be collected for ineligible participants.

# 3. INTERVENTION

Once a patient is identified and provides informed consent, they will be randomly assigned to receive either 1 dose of IV antibiotics followed by 2 days oral antibiotics or 3 doses of IV antibiotics.

***Treatment location***

Ideally, enrolment and randomisation should occur prior to the first dose or during the admission process, but the first dose of antibiotics should not be delayed by randomisation and will commence in ED. The child will either be admitted to the ward or transferred to Hospital-in-the-home (HITH) or an equivalent home-based care programme, as per routine practice. At any point from enrolment into the trial, a HITH or home-based care transfer can occur i.e., immediately after a single dose in ED or after initial admission onto the ward.

Patients randomised to 1 dose may also be admitted to the ward for observations with the cannula left in-situ or removed. This decision of treatment location is made by the treating team (ED or general paediatric) with the patient and their family as per routine clinical practice and has no effect on patient randomisation or enrolment into the trial. The treating doctor in the hospital or HITH will be responsible for the child’s clinical care throughout admission to discharge.

***Oral antibiotics switch***

Within 12 hours of the last IV dose, the patient will be switched to oral antibiotics for the remainder of their 7-day antibiotic treatment course *(cephalexin or trimethoprim/sulfamethoxazole, depending on availability at participating sites and susceptibility testing*). Patients who receive 1 dose of IV antibiotics will receive 6 days of oral antibiotics. Patients who receive 3 doses of IV antibiotics will receive 4 days of oral antibiotics. If a child vomits within 30 minutes of receiving an oral antibiotic, as per routine clinical advice a repeat dose is indicated.

***Intramuscular (IM) route***

If the IV cannula stops working or is unable to be sited before the intervention is complete in either arm, the treating clinician will decide whether there is clinical indication to re-site the cannula. If a decision is made to not re-site the cannula to complete the intervention, then the patient can be given a dose of antibiotics intramuscularly (IM). If this occurs this will not be a protocol violation and the intervention is presumed to be the appropriate treatment.

If the urine culture subsequently shows a pattern of resistance, the participant will be switched to the appropriate antibiotics by the treating clinician, which may extend IV antibiotic duration. If the duration of IV antibiotics then differs to the arm assigned, this will be recorded as a protocol violation.

***Antibiotic timing***

Antibiotic timing for the 3 doses arm can be modified if clinically appropriate as per local guidelines and clinical practice.

***Antibiotic choice***

This is a study about route of antibiotics, not antibiotic choice. In guidelines for UTI management, hospitals are encouraged to base choice on the susceptibility patterns of their own prevailing bacteria. To make the outcomes of this study as inclusive and generalisable as possible, we have ensured that the element of choice remains within the treating hospital’s guidelines. The vast majority of UTI are caused by Gram negative bacteria, followed far behind by Enterococcus spp. Some hospitals choose to empirically cover both Gram negative bacteria and Enterococcus spp; some choose only to cover for Gram negative bacteria. We have allowed for both of these practices.

**Treatment arms**

The trial will comprise two arms (Figure 2).

Arm 1, **1 dose**:

- Single dose IV to cover Gram negative bacteria followed by 2 days of oral antibiotics
- +/-
- Single dose IV to cover Enterococcus spp

Arm 2, **3 doses**:

- 3 doses IV to cover Gram negative bacteria
- +/-
- 3 days IV antibiotics to cover Enterococcus spp

In both arms, if a Gram negative organism is cultured Enterococcus antibiotic cover will cease. If Enterococcus is cultured, the Gram negative antibiotic cover will cease. If neither is cultured, the hospital will continue Gram negative cover and follow its usual practice for continuing Enterococcus cover or not. Both arms will complete a total of 7 days antibiotics, regardless of choice.

**Figure 2. Antibiotics across each intervention arm**

# 3.1. Trial Intervention(s)

# 3.1.1. Description of antibiotics

**IV/IM antibiotics**

The type of IV antibiotics administered will be based on local guidelines (IV gentamicin or IV ceftriaxone at all participating sites). The current options for IV antibiotics are below, please note IM doses are also quoted but this is only to be used If IV administrationis unsuitable. All data in the following table are from the AMH Children’s Dosing Companion, January 2020.

| **Trade or Generic name** | Gentamicin |
| --- | --- |
| **Dosage** | Children ≤10 years old: 7.5 mg/kg (maximum dose 320 mg).  Children >10 years old: 6-7 mg/kg (maximum dose 560 mg). |
| **Administration** | IV/IM once daily.  *Note. Routinely used as the preferred IV antibiotic. Gentamicin levels are not required as treatment is planned for 48 hours or less (i.e., not more than 3 doses). However, gentamicin levels can be taken as required per local hospital protocol guidelines.* |
| **Side effects** | Nephrotoxicity (with long term use – 7 to 10 days), ototoxicity (nausea, vomiting, vertigo, nystagmus, difficulties with gait). |

| **Trade or Generic name** | Ceftriaxone |
| --- | --- |
| **Dosage** | Children 1 month to 18 years old: 50-75 mg/kg (maximum dose 2 g). |
| **Administration** | IV/IM once daily.  *Note*. *Centres may use ceftriaxone instead of gentamicin per local hospital guidelines.* |
| **Side effects** | Diarrhoea, nausea, vomiting, pain and inflammation at injection site, rash, headache, dizziness, allergy. |

| **Trade or Generic name** | Amikacin |
| --- | --- |
| **Dosage** | Children ≤10 years old: 22.5 mg/kg (maximum dose 1.5 g).  Children >10 years old: 18 mg/kg (maximum dose 1.5 g). |
| **Administration** | IV/IM once daily.  *Note. Predominantly used when there is resistance to gentamicin* |
| **Side effects** | Nephrotoxicity (with long term use – 7 to 10 days), ototoxicity (nausea, vomiting, vertigo, nystagmus, difficulties with gait). |

| **Trade or Generic name** | Benzylpenicillin |
| --- | --- |
| **Dosage** | Birth (at term) – 1 week, IV 60 mg/kg every 12 hours.  1 week – 1 month, IV 60 mg/kg every 6–8 hours.  1 month – 18 years, IV/IM 30 mg/kg (maximum 1.2 g) every 6 hours. For severe infections, use up to 60 mg/kg (maximum 2.4 g) every 4–6 hours. |
| **Administration** | IV/IM |
| **Side effects** | Diarrhoea, nausea, superinfection (including candidiasis) especially during prolonged treatment with broad-spectrum penicillins, allergy. |

| **Trade or Generic name** | Amoxycillin |
| --- | --- |
| **Dosage** | Birth (term) – 1 week: 25–50 mg/kg every 12 hours  1 week – 1 month: 25–50 mg/kg every 8 hours  1 month – 18 years: 25 mg/kg (maximum 1 g) every 8 hours. For severe infections, 50 mg/kg (maximum 2 g) every 4–6 hours can be used. |
| **Administration** | IV/IM |
| **Side effects** | Diarrhoea, nausea, pain and inflammation at injection site, superinfection (including candidiasis) especially during prolonged treatment with broad-spectrum penicillins, allergy. |

*A single IM injection will not be a first line option due to the limitation in concentration of gentamicin 40 mg/ml, 1 ml max per IM injection dose, which would result in children >6 kg requiring 2 or more IM injections. However, in some situations where it is impossible to obtain IV access this will be documented in the notes and allowed for the purposes of this study and the clinical care of the patient.*

**Oral antibiotics**

The type of oral antibiotic administered will be based on clinical features of the patient’s condition and local guidelines. The current options for oral antibiotics are below (however if below oral antibiotics not suitable/unavailable the treating clinician will prescribe an alternative antibiotic and document the antibiotic name, dose, and duration in medical records).

| **Trade or Generic name** | Trimethoprim |
| --- | --- |
| **Dosage** | Children 1 month to 12 years: 3-4 mg/kg (maximum dose 150 mg). |
| **Administration** | Oral. Twice daily.  *Note*. Only tablet generally available. |
| **Trade or Generic name** | Trimethoprim |
| **Dosage** | Children 12-18 years: 300mg once daily. |
| **Administration** | Oral. Twice daily.  *Note*. Only tablet generally available |

| **Trade or Generic name** | trimethoprim/sulfamethoxazole |
| --- | --- |
| **Dosage** | Children >6 weeks old: (8mg/40mg per ml) 1ml/kg (maximum dose 40ml) |
| **Administration** | Oral. Twice daily. |

| **Trade or Generic name** | cefalexin |
| --- | --- |
| **Dosage** | 12.5-25mg/kg (maximum dosage 500mg) 4 times a day (AMH)  25mg/kg (maximum dosage 1000mg) 4 times a day (in serious infections) (AMH)  OR  *33 mg/kg (max 500 mg) oral bd  *45 mg/kg (max 1.5 g) oral tds |
| **Administration** | Oral.  *Note*. Administered if patient is allergic to trimethoprim / sulfamethoxazole.  *This dosing is from the RCH Clinical Practice Guidelines |

| **Trade or Generic name** | cefuroxime |
| --- | --- |
| **Dosage** | 3 months – 2 years, 10 mg/kg (maximum 125 mg) twice daily.  2–12 years, 15 mg/kg (maximum 250 mg) (up to 500 mg twice daily has been used).  12–18 years, 250–500 mg twice daily. |
| **Administration** | ORAL every 12 hours. |

NB: Please note the inclusion age for participants for this study is **3 months (corrected age) to 18 years (<19 years)**. To ensure accurate referencing of AMH Children’s Dosing Companion some of the above doses have been stated from 1 month.

# 3.1.2. Measurement of participant compliance

Compliance with treatment will be assessed in all patients. Evidence of administration of IV antibiotics will be automatically documented on a drug chart as per routine clinical practice.

Compliance and non-compliance with oral antibiotics will be recorded for study purposes, where it will be used to evaluate the total number of days antibiotics were administered. However, it will not impact on patient inclusion in each randomisation arm.

# 3.1.3. Excluded medications and concomitant therapy

Not applicable to this trial.

# 4. RANDOMISATION

In the Emergency Department, if the patient is identified as appropriate for the study they will then be randomly assigned to receive treatment arm 1 or treatment arm 2. Randomisation will be performed through web-based randomisation or envelopes by a member of the research team (study investigators or trained clinicians) at the time of receiving the first dose of IV antibiotics or when the ED is extremely busy, the admission process by the ward clinician. Randomisation will have no effect on whether they are admitted to the ward or HITH. Once a patient has been assessed in the ED as per routine practice there will be a discussion between the clinician and the family about whether this patient can be managed on HITH or if they should be admitted to the ward.

A randomisation schedule will be prepared by a statistician independent from the study in the Clinical Epidemiology and Biostatistics Unit (CEBU) at the Murdoch Children’s Research Institute. The randomisation schedule will have an allocation ratio of 1:1 for each treatment arm and will be assigned in randomly permuted blocks of size 2,4, and 6, stratified by age (<1 year versus ≥1 years), number of complicated factors (<=3 factors versus 4 or more factors), and 5 sites.

TRIAL VISITS AND PROCEDURES

# 4.1. Schedule of assessments

Table 4. Schedule of assessments

|  | **Day 1** | **Day 2** | **Day 3** | **Day 4** | **Day 5-7** | **Day 14** | **1 month** |
| --- | --- | --- | --- | --- | --- | --- | --- |
| **TIME POINT*** | **ED presentation (1^st^ dose of IV antibiotic)** | **24h** | **48h** | **72h** |  |  |  |
| **ENROLMENT:** |  |  |  |  |  |  |  |
| **Eligibility screen** | X |  |  |  |  |  |  |
| **Informed consent** | X |  |  |  |  |  |  |
| **Allocation to intervention** | X |  |  |  |  |  |  |
| **INTERVENTIONS:** |  |  |  |  |  |  |  |
| **Arm 1: One dose** | IV | oral | oral | oral | oral |  |  |
| **Arm 2: Three doses** | IV | IV | IV | oral | oral |  |  |
| **ASSESSMENTS:** |  |  |  |  |  |  |  |
| **Demographic information ^a^** | X |  |  |  |  |  |  |
| **Clinical assessment ^b^** | X |  |  |  |  |  |  |
| **Parental assessment daily** ^c^ | X | X | X | X |  |  |  |
| **Parental cost data ^d^** |  |  |  |  |  | X |  |
| **Primary outcome** ^e^ |  |  |  | X |  |  |  |
| **Clinical assessment – other criteria** ^f^ | X | X | X | X |  |  |  |
| **Day 14 review** ^g^ |  |  |  |  |  | X |  |
| **QoL survey** ^h^ | X |  |  | X |  | X |  |
| **Final review ^i^** |  |  |  |  |  |  | X |

Notes.

* Timepoint refers to the time lapsed from the initial 1^st^ dose of IV antibiotic. Ideally, each assessment will be conducted prior to the next antibiotic dose.

^a^ Patient demographic information will be collected from the patient’s clinical notes. This will include: date of birth, gender, and postcode.

^b^ Clinical information other than complicated features performed by the treating medical doctor or nurse, which is usually part of routine care. Clinical information will be collected to facilitate evaluation of outcomes. This will include presence of: lethargy, abdominal pain, flank pain/tenderness, dysfunctional voiding, dysuria, offensive urine, gross haematuria, frequency, suprapubic tenderness, antibiotic use (prophylaxis), known resistant organisms. Method of urine collection in ED will be documented.

^c^ Daily parental assessment: In both arms, parents will be asked to prospectively record presence of fever, vomiting, rigors, and tachycardia in a diary which will be provided at enrolment. Hospital grade thermometers will be provided for families. Parents will be shown how to use thermometers and advised to record symptoms 3 times a day (morning, afternoon, night) without pre-determined times.

^d^ Parental cost data collection: In both arms, parents will be asked to report in the previous 14 days since the first dose of antibiotic – leave taken for paid and unpaid work, extra expenditure related to the child’s illness such as medication, transport, accommodation, childcare, food, collected at Day 14.

^e^ Primary outcome – Clinical failure defined as *persistence of baseline symptoms* (**fever or vomiting or rigors**) or *development of new symptoms* (**fever or vomiting or rigors**) *attributable to UTI*: at 72 hours assessed at least 6 hours after last antipyretic. Presence of **fever, vomiting, rigors** reported by parents within 6 hours of the 72 hour assessment will be recorded as present at assessment. If fever persists will advise to present to GP or contact the general paediatrician or re-present to ED.

^f^ Other clinical information will be collected to facilitate evaluation of outcomes. Review of clinical interventions during admission (therapy prior to discharge) will include: IV or nasogastric fluids, antiemetic medications, adverse events, allergic reactions, pain relief medications (paracetamol, ibuprofen) and complications.

The Day 14 review will be conducted over phone or telehealth.

^h^ The Child Health Utility 9D (CHU9D) questionnaire will be used to evaluate health related quality of life for children. The CHU9D will be administered to children aged 7-17 years for completion via self-report (7-11 years, 11-17 years). Proxy versions will be administered to the parent/guardian to complete for children less than 7 years (5-7 years, <5 years).(24) In addition, the EQ-5D will also be administered to measure parent quality of life. Both the questionnaires are validated.

^i^ The 1-month review will be conducted by text message initially for participant convenience (Appendix). If the participant reports recurrence of UTI or hospital admission for UTI, they will be contacted by phone or telehealth for further details. If we do not receive replies to the text message, they will be contacted by phone or telehealth.

# 4.2. Notes on specific trial visits

# 4.2.1. Screening

Prior to trial commencement, clinicians will be informed of the trial and eligibility criteria via online or in person education sessions delivered by the research team (Refer to Section 4.6). This will ensure ED clinicians are familiar with the eligibility criteria for inclusion and exclusion of potential trial participants.

In the ED, clinicians will screen all patients presenting to the ED with suspected UTI against the eligibility criteria (diagnosis, age, complicated features). Any uncertainty with patient eligibility will be discussed with a member of the research team.

Patients who are screened as eligible will be provided with information describing the study and invited to participate. This will include a brief summary of the trial and a copy of the Information and Consent Form. During this time, the patient and parent/guardian will be given the opportunity to ask questions about the trial. If they are willing to participate, they will be asked to sign the consent form in person or electronically. Once they have provided informed consent, the participant will be randomised to a trial intervention.

As per routine clinical practice, the treating team will be responsible for following the urine culture results and ensuring the antibiotics prescribed are appropriate. Resistance of bacteria grown in initial urine culture to the study antibiotics is addressed under 7.3.5 Continuation of therapy and Deviations.

# 4.2.2. Final trial visit

The final trial assessment will occur 1 month after randomisation, participants will be offered by telephone or telehealth or in person by a member of the research team.

Optional consent will be requested to contact participants in the future for related research studies. This will be included in the Information and Consent Form.

# 4.2.3. Unscheduled visit

We do not anticipate the need for any unscheduled visits. However, if these are required for safety review or other reason, they will be discussed with senior investigator and clinical team and handled as per RCH guidelines. All unscheduled visits will be documented in the patient’s research file.

# 4.3. Participant withdrawal, replacement and losses to follow-up

# 4.3.1. Withdrawal of consent

Participants will be informed at the time of obtaining consent that they may withdraw from the study at any stage. Withdrawing from the trial will not affect their access to treatment or management of their medical condition or their relationship with the hospital or treating team.

If participants express their desire to withdraw from the study, 2 options will be discussed: i) withdrawal from treatment but continue to participate in the study and data will continue to be collected; or ii) withdraw from treatment and study whereby no more data or samples will be collected. Data and samples collected prior to withdrawal of consent will be retained, unless the patient or parent/guardian requests otherwise.

# 4.3.2. Losses to follow-up

A participant will be considered lost to follow-up if he or she fails to complete the Day 14 assessment and/or the final assessment (1 month) and is unable to be contacted by the trial site staff.

The following actions must be taken if a participant fails to complete assessments:

- The research team will attempt to contact the participant within 1 week of the scheduled final assessment.
- If follow up surveys are not completed at 14 days and 1 month, follow up questions in the ‘Adverse Events’ and ‘Protocol Deviation’ REDCap surveys can be completed by the research team using the medical notes (if available).
- 72-hour follow up must be completed. If the 72-hour follow up is not completed by parent/guardian, the research team can complete using the medical notes (if available).

# 4.3.3. Replacements

Withdrawals will not be replaced. Patients who are found to not meet the inclusion/exclusion criteria after being randomised (e.g., randomised but ineligible due to culture results and/or radiological evidence not consistent with UTI) will be replaced by further recruitment to maintain the required sample size. The urine culture results confirming a UTI will not be available until 48 to 72 hours after sample collection. Only after this is available will the patient be definitively included in the population to analyse. Patients who do not meet culture or radiological confirmation but has a discharge diagnosis and clinical management of presumed UTI (i.e., the treating clinician has provided a diagnosis of UTI and treated the patient accordingly) will also be included in the population to analyse.

# 4.3.4. Trial Closure

A participant is considered to have completed the trial if he or she has completed all phases of the trial including the final assessment at 1 month (after the initial IV dose) (Table 3, Section 7.1).

When the study is closed, results analysed and manuscripts published, a letter will be written to all participants thanking them for their involvement. This letter will also include a summary of the trial highlighting the main outcomes from the trial. The letter will be mailed to their residential address located on their clinical file in EMR. The data will be presented as aggregate data, where no individual participant will be identified.

# 4.3.5. Continuation of therapy

Continuation of therapy beyond day 7 post first dose IV antibiotics, (IV or oral antibiotics) will be at the discretion of the patient’s clinical team. If this occurs, details of the therapy and duration will be documented in the patient’s research file. Deviation from the assigned antimicrobial treatment regimen or not adhering to the pre-determined duration of IV antibiotics will be deemed as a violation in the following events: (i) clinical deterioration as assessed by the treating clinician (e.g., clinical suspicion of abscess, evolving sepsis or meningitis); or (ii) presumed side effects of the study drugs; or (iii) resistance of bacteria grown in initial urine culture to the study antibiotics necessitating change in empiric treatment and non-adherence to duration of IV treatment; or (iv) patient unable to tolerate oral antibiotics.

# 5. SAFETY EVENTS AND RISKS

# 5.1. Adverse events – definitions

Adverse event: Any untoward medical occurrence to a participant enrolled in this study regardless of its causal relationship to study treatment.

Serious adverse event:

Adverse events are classified as serious or non-serious. A SAE is defined as any AE that:

Results in death; or

Is immediately life-threatening; or

Requires inpatient hospitalisation; or

Results in prolongation of existing hospitalisation; or

Results in persistent or significant disability/incapacity

Significant medical events will be considered a SAE when, based upon appropriate medical judgement, they may jeopardise the participant and may require medical or surgical intervention to prevent one of the outcomes listed above.

Suspected Unexpected Serious Adverse Reaction (SUSAR):

A SUSAR is any SAE that which there is some degree of probability that the event is related to the study drug and the adverse reaction is unexpected (that is, not consistent with the applicable product information).

Significant Safety Issues (SSI):

A SSI is a safety issue that could adversely affect the safety of participants or materially affect the continued ethical acceptability or conduct of the trial.

For the purposes of this study the research team is responsible for recording in the patient’s research record and following up on all adverse events (AE) and adverse reactions (non-serious and serious) from the time of the initial IV dose until Day 14. For the purposes of this study the investigator or delegate will be responsible for recording AEs regardless of their relationship to the study drug, with the following exceptions:

Conditions that are pre-existing at enrolment and do not deteriorate will not be considered AEs.

Abnormal laboratory values will not be considered AEs unless deemed clinically significant by the investigator and documented as such.

# 5.2. Documentation of adverse events

Adverse event information will include (this will be documented in the patient’s clinical file by the research team):

- A description of the adverse event
- The onset date, duration, date of resolution
- Severity (mild, moderate, or severe)
- Seriousness (i.e., is it a serious adverse event?)
- Any action taken, (e.g., treatment, follow-up tests)
- The outcome (recovery, death, continuing, worsening)
- The likelihood of the relationship of the adverse event to the trial treatment (Unrelated, Possible, Probable, Definite)

Adverse events will be recorded at the 72-hour assessment as well as at the Day 14 follow up assessment for all participants. If the AE is deemed clinically relevant, the treating team will be informed. For hospital ward patients it will be the treating medical team; for patients being treated at home this will be the HITH consultant and registrar.

Serious AE (SAE) such as overwhelming sepsis or death are not expected in this study as these patients will have been excluded from the outset. If it occurs, sepsis will be considered an SAE. All unanticipated AEs (including SAEs) will be documented with a full description in the study records and on the case report form. AEs that occur within the timeframe as described above will be followed until either resolution or hospital discharge, whichever occurs first.

If an SAE has not resolved at the time of the initial report and new information arises that changes the investigator’s assessment of the event, a follow-up report including all relevant new or reassessed information (e.g., concomitant medication, medical history) will be submitted to the local HREC. The investigator is responsible for ensuring all SAE are followed until either resolved or stable.

# 5.3. Assessing the seriousness of a participant’s adverse event

The seriousness of an adverse event will be assessed by an investigator according to the definition in the preceding section on definitions with the following exception(s):

- Hospitalisation due to progression of disease will not be considered a SAE/Serious Adverse Reaction (SAR) for the purposes of this trial.
- Elective surgery planned at the time of enrolment will not be considered a SAE for the purposes of this trial.
- The seriousness of an adverse event will be assessed by an investigator according to definitions listed in NHMRC Guidance: Safety monitoring and reporting in clinical trials involving therapeutic goods (dated November 2016) <https://www.nhmrc.gov.au/guidelines-publications/eh59>

# 5.4. Reporting of safety events

Site Principal Investigator Reporting Procedures

The Site Principal Investigator/delegate is responsible for recording all safety events in the clinical report forms. The Investigator is responsible for expedited reporting (within 24 hours of becoming aware of the event) to the Sponsor-Investigator the following local safety events:

1. Urgent Safety Measures (USMs)
2. Suspected Unexpected Serious Adverse Reactions (SUSARs)
3. All SAEs /SARs, except those that are identified above as expected in the trial population:

The Site Principal Investigator is responsible for reporting SAEs (including SUSARs) to the Sponsor-Investigator as soon as possible but within 24 hours of the first knowledge of the event. These reports should be submitted using the trial Expedited Safety Report Form.

The Site Principal Investigator is also responsible for reporting Significant Safety Issues (SSIs), local USMs and local SUSARs to their research governance office within 72 hours of becoming aware of the event and in accordance with their local governance authorization.

Sponsor-Investigator Reporting Procedures

The Sponsor-Investigator must assess and categorise the Expedited Safety Reports received from Investigators and report these to all Site Principal Investigators, the approving HREC and TGA in accordance with the NHMRC’s ‘Safety monitoring and reporting in clinical trials involving therapeutic goods’ (November 2016) and any additional requirements of the approving HREC. All safety reports must clarify the impact of the safety event on participant safety, trial conduct and trial documentation.

The Sponsor-Investigator is responsible for the following reporting to Pis, the HREC(s) and TGA:

1. All SSIs that meet the definition of a USM within 72 hours of becoming aware of the issue.
2. All other SSIs within 15 calendar days of instigating or becoming aware of the issue
3. For SSIs leading to an amendment of trial documentation:
   a. Submit details of the SSI without undue delay and no later than 15 calendar days of becoming aware of the issue.
   b. Submit amendment to the HREC without undue delay.
4. For SSIs leading to temporary halt or early termination of a trial for safety reasons:
   a. Communicate reasons, scope of halt, measures taken, further actions planned without undue delay and no later than 15 calendar days of decision to halt.
   b. For a temporary halt, notify the Pis, HREC and TGA when the trial restarts, including evidence that it is safe to do so.

The Sponsor will also report SUSARs to the TGA as follows:

1. Fatal or life-threatening SUSARs immediately, but no later than 7 calendar days after being made aware of the issue (follow up info within a further 8 calendar days)
2. All other SUSARs no later than 15 calendar days of being made aware of the issue

The Sponsor is responsible for providing the additional safety information to the approving HREC:

1. Provide an annual safety report, including a summary of the evolving safety profile of the trial.
2. Provide any updated Product Information/Investigator’s Brochure for the investigational products (if applicable).

The Sponsor is also responsible for providing any updated Product Information/Investigator’s Brochure to Investigators.

# 6. DATA MANAGEMENT

# 6.1. Data Collection, processing, and storage

The Principal Investigator is responsible for storing essential trial documents relevant to data management and maintaining a site-specific record of the location(s) of the site’s data management-related Essential Documents.

The Principal Investigator is responsible for maintaining adequate and accurate study documents that include all key observations on all participants at their site. Study data will be attributable, legible (including any changes or corrections), contemporaneous, original, accurate, complete, consistent, enduring, and available. Most study data is collected through medical chart review and therefore will contain little source data.

The Principal Investigator will also maintain accurate case report forms (CRFs) (i.e., the data collection forms) and be responsible for ensuring that the collected and reported data is accurate, legible, complete, entered in a timely manner and enduring. To maintain the integrity of the data, any changes to data (hardcopy and electronic) must be traceable, must not obscure the original entry, and must be explained where this is necessary.

Any person delegated to collect data, perform data entry or sign for data completeness will be recorded on the delegation log and will be trained to perform these trial-related duties and functions.

Full details of all processes are provided in a separate trial-level **Manual of Operations.**

# 6.1.1. Source Data

Data will be collected from the patient’s medical record; participant- or researcher-completed questionnaires or rating scales (e.g., CHU9D).

# 6.1.2. Unique participant identifier

All study participants will be assigned a unique study identifier (Participant ID) for the trial. The document linking the Participant ID with personal identifiers will be password protected and accessible only to the researchers. The unique study identifier will be used to identify all related study documents across research databases.

# 6.1.3. Data Capture Methods and Storage

Patient/participant identifiable electronic data of consented and non-consented (screened, but not enrolled) will be stored on individual site password-protected databases located on a secure computer within each site. This screening database will only be accessed by members of the local research team. The screening database will store identifiable participant information about screening, consent, enrolment, and withdrawal. For all participants (consented and non-consented) it will contain the participant’s name along with their unique ID number. Detailed information about recruitment success and failure will be collected for each patient in the screening log. Participants who decline to participate will not be required to provide a reason for their decision. However, if they voluntarily provide a reason for refusal, we will record the reason in their research file. Hard copy patient/participant identifiable consent forms will be stored in locked storage in the Emergency Research office or other site as applicable.

For the purposes of randomisation, data accuracy, and follow up, the patient’s hospital number and contact details will be entered in REDCap during data collection. These will be removed from the database when data collection finishes. Only re-identifiable research data of consented participants will be stored in REDCap. The participant research record will be linked to the participant using a unique study identifier. The study REDCap database is hosted on MCRI infrastructure and is subject to the same security and backup regimen as other systems (e.g., the network file servers). Data is backed up nightly to a local backup server, with a monthly backup taken to tape and stored offsite. All data transmissions between users and the REDCap server are encrypted. Regular data quality checks, such as automatic range checks, will be performed to identify data that appear inconsistent, incomplete, or inaccurate.

Access to REDCap is via a MCRI user account or (for external collaborators) via a REDCap user account created by the system administrator. The permissions granted to each user within each REDCap project is controlled by and is the responsibility of the project team. Staff at individual sites will only have access to their local data. The trial CPI and trial co-ordinator will be responsible for overseeing the data collection and will have access to the complete study database. REDCap has functionality that makes adding and removing users and managing user permissions straightforward. REDCap maintains an audit trail of data create/update/delete events that is accessible to project users that are granted permission to view it.

# 6.1.4. Record Retention

Data will be kept for at least 15 years after the completion of a clinical trial in accordance with the requirements of the Therapeutic Goods Administration or until the 25th birthday of the youngest participant (Victorian Health Records Act 2001) (whichever is the later date).

# 7. STUDY OVERSIGHT

# 7.1. Governance structure

Appropriate oversight of trial conduct, protocol compliance, safety and data integrity will be the responsibility of the site PI. The site PI will review the data for safety concerns and data trends at regular intervals and will promptly report to the HREC and the central coordinating team any significant protocol deviation or any other significant event or problem that arises during the conduct of the study.

# 7.1.1. Trial Management Group (TMG)

The Principal Investigator is responsible for supervising any individual or party to whom they have delegated tasks at the trial site. They will provide continuous supervision and documentation of their oversight. A small group will be responsible for the day-to-day management of the trial and will include the principal investigator and project manager/research nurse/trial coordinator. The group will closely review all aspects of the conduct and progress of the trial, ensuring that there is a forum for identifying and addressing issues. Meetings will be minuted with attendees listed, pertinent emails retained, and phone calls documented.

# 7.1.2. Trial Steering Committee (TSC)

A Trial Steering Committee will be established to provide expert advice and overall supervision and ensure that the trial is conducted to the required standards. The Committee will meet at least annually, with more frequent meetings as needed.

# 7.1.3. Safety Monitoring

Safety oversight will be under the direction of an independent Data and Safety Monitoring Board (DSMB). It will be composed of individuals with the appropriate expertise, including at least three independent clinicians and/or biostatisticians who, collectively, have experience in the management of paediatric patients, biostatistics and the conduct and monitoring of randomised controlled trials. Members of the DSMB will be independent of trial conduct. The DSMB will meet within 12 months after commencing the trial, and annually for the duration of the trial. At each meeting, a descriptive summary of status of accrual, reasons for not randomising, withdrawals, protocol violations, serious adverse events and non-serious adverse events thought to be related to the study drugs will be provided.

The DSMB will review data on safety, feasibility, and protocol violations to subsequently inform the sponsor or TSC on whether to continue, modify or stop a trial for safety or ethical reasons.

The DSMB will be responsible for safeguarding the interests of trial participants, monitoring the safety of the trial interventions and reviewing protocol non-compliance. To contribute to enhancing the integrity of the trial, the DSMB may also formulate recommendations relating to the selection, recruitment and retention of participants, participant management, improving adherence to the protocol and the procedures for data management and quality control. The DSMB will provide input and report to the PI.

The DSMB will agree on a charter detailing its role and procedures to ensure transparency of decisions made to the Sponsor-Investigator.

# 7.2. Quality Control and Quality Assurance

The trial investigator/trial coordinator will develop SOPs that identify, evaluate and control risk for all aspects of the trial, e.g., trial design, source data management, training, eligibility, informed consent, and adverse event reporting. These can be located in the Manual of Operations (MOO). The trial coordinator will implement quality control (QC) procedures, which will include the data entry system and data QC checks. Any missing data or data anomalies will be communicated to the principal investigator for clarification/resolution.

Quality Management is the overall process of establishing and ensuring the quality of processes, data, and documentation associated with clinical research activities. It encompasses both quality control (QC), and quality assurance (QA) activities. Both the Sponsor-Investigator and Site Investigator have responsibilities in relation to quality management.

As outlined in the previous section (Site Monitoring), the trial monitors will verify that the clinical trial is conducted and data are generated, biological specimens are collected, documented (recorded), and reported in compliance with the protocol, good clinical practice, and applicable regulatory requirements.

In the event of non-compliance that significantly affects human participant protection or reliability of results, the investigator/trial coordinator will perform a root cause analysis and corrective and preventative action plan (CAPA).

# 8. STATISTICAL METHODS

# 8.1. Sample Size Estimation

Data on clinical failure at 72 hours for children who receive 1 dose of IV antibiotics in this cohort is lacking but estimated to be similar to 3 days of IV antibiotics due known high efficacy of oral antibiotics. We prespecified a non-inferiority margin for 1 dose of IV antibiotics of 10 percentage points above the proportion of children with clinical failure at 72 hours in the 3 doses IV antibiotics group for which the assumption is 5%. 1 dose of IV antibiotics would thus be considered non-inferior to 3 days of IV antibiotics if the upper limit of the two-sided 95% confidence interval for the risk difference was less than 10 percentage points. For 85% power and an assumption of 1 dose of IV antibiotics to have a failure rate of 7%, a sample size of 316 children is required (two-sided alpha level of 0.05). A 20% rate of loss to follow-up or protocol violations is expected, therefore 396 children will be recruited, i.e., 198 in each arm.

The expected number of children fulfilling the inclusion criteria of a UTI with complicated features will be around 50 per year at the RCH. Including the 5 PREDICT network across Australia, (a well-established collaboration of paediatric ED clinician-researchers) the number of expected children fulfilling inclusion criteria would be 594 per year. Accounting for a conservative 40% recruitment rate, recruitment is expected to take at least 2 years from when all sites are actively recruiting.

# 8.2. Justification of Non-Inferiority Margin

Consistent with the literature, this non-inferiority was by consultation with i) clinicians/investigators, all of whom represent different areas of practice, ii) a consumer representative, a parent of a child previously treated with IV antibiotics and iii) a review of the literature:

i. From the investigators (with representation from various groups – Infectious Diseases, General Paediatrics, Emergency and Nephrology clinicians).

This non-inferiority margin of 10% was chosen because the expected gain from reducing the use of IV antibiotics is important. If there was a risk that the infection could have severe outcomes, the margin would have to be smaller because any advantages of the shorter treatment course would not be offset by an unacceptable increase in serious outcomes.

In our proposed study, failure only means that it takes longer than 72 hours for a child to ‘feel better’ i.e., resolved fever, no vomiting and no rigors. Failure does not equate to disability or death.

We estimate 5% of children treated with 3-day IV treatment, to fail and take longer than 72 hours to clinically improve. As above, we would accept a 10% difference of clinical failure.

From a clinicians’ perspective, if in the 1 dose arm, 15% of children take longer to get better (1-2 days more of fever) but it still means 85% of children avoid all the adverse effects of receiving 3 days of IV antibiotics i.e., longer hospital stay (hospital acquired infections, drug errors), side effects of antibiotics (repeated IV drip insertion, diarrhoea) and overall improved quality of life, this is an acceptable margin.

ii. The consumer representative who was the parent of a child previously treated with IV antibiotics for UTI and whose child had multiple admissions to hospital had input in the design of the study, specifically in the choice of the primary outcome. This consumer was consulted on the acceptable difference in margin, and she agreed that this margin is acceptable.

iii. The following table lists previous trials on UTI and other childhood infections in the literature where a 10% margin or more was accepted, in some cases with more severe outcomes:

| **Title, Author, Journal** | **Non-inferiority margin,**  **Primary outcome** | **Infection type, Details of sample size calculation** |
| --- | --- | --- |
| Antibiotic treatment for pyelonephritis in children: multi-centre randomised controlled non-inferiority trial. Montini et al. BMJ | 10%  Kidney scars | UTI Children. In around 15% of children with acute pyelonephritis treated with parenteral antibiotics, scintigraphy 12 months after infection shows  measureable renal scars. We therefore considered that an upper confidence limit of 25% for the new (oral) treatment would indicate non-inferiority. As previously suggested we adopted 10% as the margin of equivalence. |
| Randomised trial of oral versus sequential intravenous/oral  cephalosporins in children with pyelonephritis. Neuhaus et al. Eur J Pediatrics | 20%  Kidney scars | UTI Children. On the basis of our previous study, we estimated that the incidence of renal scarring was approximately 35%. This is a non-inferiority trial where a maximal difference of 20% between the percentages of renal scarring of the two groups is considered to be acceptable.  For a power of 90% and a type 1 error level of 5% (two tailed), the sample size should be 98 children completing the study in each group; for a power of 80%, the sample size should be at least 71 children in each group. |
| Oral Versus Initial Intravenous Therapy for Urinary Tract Infections in Young Febrile Children. Hoberman et al. Pediatrics | 15%  Kidney scars | UTI Children. It was estimated that 30% of children with UTI treated with IV antimicrobials would have renal scarring of some degree at 6 months.  To detect an absolute difference of 15% in the incidence of renal scarring between children treated orally (45%) compared with those treated intravenously, at a value of .05 (one-tailed) and with a power of .80, 128 children per treatment group were needed. |
| Once-daily plazomicin for complicated urinary  tract infections. Wagenlehner et al. NEJM | 15%  Resolution of symptoms and microbiological response | UTI Adults. A sample size of 394 patients in the mMITT population provides at least 85% power to demonstrate non-inferiority of plazomicin to meropenem with a 15% non-inferiority margin and a one-sided significance level of 0.025 for both the co-primary endpoints, assuming a response rate in both treatment groups of 64% at Day 5and 73.2% at test of cure. |
| Ciprofloxacin for 7 days versus 14 days in women with acute pyelonephritis: a randomised, open-label and double-blind, placebo-controlled, non-inferiority trial. Sandberg et al. Lancet. | 10%  Resolution of symptoms | UTI Adults. The size of the study sample was calculated on the basis of a cure rate of 10 percentage points lower at short-term follow-up with 7 days of therapy, with the assumption of a 92% cure rate in patients treated for 14 days.  The chosen margin of non-inferiority is quite wide because the expected gain from reducing the use of antibiotics is important. If there was a high risk that the infection could be life-threatening after the first week of treatment, the margin would have to be smaller because any advantages of the shorter treatment course must not be offset by an unacceptable increase in serious deterioration.  The required sample size per group for a non-inferiority test for two binomial populations, with type 1 error 0·05, power 0·8, and non-inferiority margin 0·10, is 90 by use of the Wald test.  Assuming that an estimated 30% of the randomly assigned patients would for various reasons be excluded from the efficacy analysis, we planned to recruit about 260 patients to the study. |
| Shortened Antimicrobial Treatment for Acute Otitis Media in Young Children. Hoberman et al. NEJM | 10%  Resolution of symptoms | Ear infection children. We estimated that among children receiving 10-day antimicrobial treatment, 15% would have clinical failure, and we specified that the 5-day treatment would be considered to be non-inferior (and accordingly, clinically acceptable) if the upper boundary of the 95% confidence interval of the between-group difference in the percentage of children with clinical failure (percentage in the 5-day group minus the percentage in the 10-day group) would be no greater than 10 percentage points.  Assuming clinical-failure rates of 15% in the 10-day group and 25% in the 5-day group, statistical power of 0.95, and a 10% rate of loss to follow-up or withdrawal from the study, we calculated that the study would need to include a sample of 300 children per group. |
| Efficacy and safety of intravenous ceftriaxone at home  versus intravenous flucloxacillin in hospital for children with  cellulitis (CHOICE): a single-centre, open-label, randomised,  controlled, non-inferiority trial. Ibrahim et al. Lancet ID | 15%  Resolution of symptoms | Skin infection children. Thus, for this study, we assumed that about 7% of children treated in hospital would have treatment failure and estimated that treatment failure would occur in about 10% of children treated at home.  We specified that for the home group to be non-inferior to the hospital group (and clinically acceptable based on the literature and discussions with clinicians in the emergency department), the upper margin of the two-sided 95% CI of the difference in the primary outcome between the home and hospital groups should be no greater than 15%. |

# 8.3. Population to be analysed

The following participant populations will be analysed:

- Intention-to-treat population (ITT): Includes any participant meeting the inclusion criteria, regardless of randomisation arm and regardless of whether they received the trial intervention. It will only exclude those participants who are randomised but later found ineligible due to urine results and/or radiological evidence not consistent with UTI. This exclusion does not apply where the treating clinician has provided a diagnosis of presumed UTI and managed the patient accordingly.
- Per-protocol population (PP): Includes any participant who was randomised and received the protocol-required doses of trial drug and fulfilled all protocol required assessments and did not do any major protocol violation, such as not adhering to the pre-determined duration of IV antibiotics.

# 8.4. Methods of analysis

Data analysis for the study will be performed by the Principal Investigator with CEBU oversight under the trial statistician’s supervision. The Principal Investigator and trial statistician (LI and FO) will be blinded to treatment assignment until the completion of analysis. Statistical analysis will follow both intention-to-treat (ITT) and per-protocol (PP) approaches, which is common for non-inferiority randomised controlled trials.

The ITT population will include any participant meeting the inclusion criteria, regardless of randomisation arm and regardless of whether they received the trial intervention. The modified intention to treat population will consist of all eligible randomised participants with a confirmed or presumed diagnosis of UTI (confirmed by urine culture or have radiological evidence, or presumed on the basis of treating clinician diagnosis and clinical management as UTI documented at discharge).

Baseline characteristics (such as patient demographics, underlying diagnosis, presence of systemic symptoms) will be presented separately for participants in the arms using means and standard deviations (SD) for continuous data (or medians and inter-quartile ranges for non-normal data) and proportions for categorical data.

Risk Differences (RD) between the 2 arms will be the measure of treatment effect for binary outcomes. Mean difference between treatment groups will be the measure of treatment effect for continuous outcomes whose distribution is reasonably symmetrical. Median difference between treatment groups will be the measure of treatment effect for continuous outcomes whose distribution is severely skewed.

Treatment effects will be estimated using regression methods (generalised linear models, GLM, or quantile regression models) to adjust for the stratification factors and in secondary analyses for additional covariates that are expected to be associated with the primary outcome (urological abnormalities, history of recurrent UTI, fever).

*Primary analysis*

The number and proportion of participants who will be deemed as clinical failures by the treating clinician within 72 hours from the first dose will be summarised by treatment arm, using absolute and relative frequencies.

The risk difference with 95% confidence internal (CI) will be estimated using a generalised linear model (GLM) to adjust for the strata used during randomisation as a covariate, age (<1 year versus ≥1 years), number of complicated factors (3 and under factors versus 4 or more factors), and site. The GLM approach will use the binomial family and employ an identity link function. In case of a high proportion of treatment crossover in the study, which is likely to attenuate the estimated effect of the intervention, secondary analysis on the primary outcome will be conducted to account for treatment crossover using inverse probability weighting (IPW) techniques.

We have pre-specified the margin of non-inferiority for 1 dose treatment as 10% above the 3 days treatment failure rate. For the 1 dose IV antibiotics to be non-inferior to 3 days antibiotics, the upper limit of the two-sided 95% CI of the risk difference must be less than 10%. For the primary outcome, the GLM specification above will be extended to include further adjustment for the following covariates, which are expected to be associated with the primary outcome: (urological abnormalities, history of recurrent UTI, fever)

Sensitivity analysis will be done including those who have been excluded due to not meeting microbiological criteria.

Stata version 16.0 or above will be used for all analyses.

A detailed Statistical Analysis Plan will be written by the study statistician ahead of the database lock and before running the analysis.

*Economic analysis*

Cost effectiveness analysis of single dose of IV followed by 2 days oral antibiotics compared to 3 doses IV will be conducted using the trial data. The costs associated with hospital stay as well as downstream healthcare costs and family out-of-pocket will be estimated for each study arm, using administrative hospital costing data and parent questionnaires.

Parents will also be surveyed for the impact of the condition on productivity loss including paid and unpaid leaves. The incremental cost per infection resolved and per quality adjusted life year (QALY) gained will be estimated for the 1 dose versus 3 doses IV arms. Extensive probabilistic and one-way sensitivity analyses will be conducted based. Cost-effectiveness analysis will be considered in the context of a non-inferiority trial.

# 9. ETHICS AND DISSEMINATION

# 9.1. Research Ethics Approval & Local Governance Authorisation

Institutional Ethics Committee approval will be sought at the Royal Children’s Hospital prior to the commencement of this project and SSA approval at participating Australian sites. Any amendments to the Protocol will be submitted to the HREC for review and approval. This protocol and the informed consent document and any subsequent amendments will be reviewed and approved by the RCH Human Research Ethics Committee (HREC). A letter of protocol approval by HREC will be obtained prior to the commencement of the trial, as well as approval for other trial documents requiring HREC review. Non-Australian sites (Starship New Zealand) will seek local Institutional Ethics Committee approval.

# 9.2. Amendments to the protocol

This trial will be conducted in compliance with the current version of the protocol. Any change to the protocol document or Informed Consent Form that affects the scientific intent, trial design, participant safety, or may affect a participant’s willingness to continue participation in the trial is considered an amendment, and therefore will be written and filed as an amendment to this protocol and/or informed consent form. All such amendments will be submitted to the HREC, for approval prior to being implemented.

# 9.3. Protocol Deviations and Serious Breaches

All protocol deviations will be recorded in the participant’s research record and will be reported to the principal investigator, who will assess for seriousness.

Deviations deemed to significantly affect the rights of a trial participant or the reliability and robustness of the data generated in the clinical trial will be reported as serious breaches. Reporting will be done in a timely manner (Trial coordinator to report to the trial investigator within 72 hours and to the Research Governance Office within 7 days; Principal Investigator to review and submit to the approving HREC within 7 days).

Where non-compliance significantly affects human participant protection or reliability of results, a root cause analysis will be undertaken, and a corrective and preventative action plan prepared. This must be submitted to the Principal Investigator within 7 days.

Where protocol deviations or serious breaches identify protocol-related issues, the protocol will be reviewed and, where indicated, amended. The minimum doses for each arm of the intervention, which would not invoke a serious breach, are as follows: one dose arm - 1 dose of IV/IM antibiotic, three dose arm - 2 doses IV/IM antibiotic (if once daily), or 5 doses if 6 hourly, or 4 doses if 8 hourly.

Deviations from the assigned antimicrobial treatment regimen or not adhering to the pre-determined duration of IV antibiotics include the following events:

(i) Clinical deterioration as assessed by the treating clinician (e.g., clinical suspicion of abscess, evolving sepsis or meningitis).
(ii) Presumed side effects of the study drugs e.g., allergic rash to ceftriaxone.
(iii) Resistance of bacteria grown in initial urine culture to the study antibiotics - treatment will then be managed according to the clinical situation.
(iv) Patient unable to tolerate oral antibiotics – persistent vomiting.

The above will constitute a protocol deviation for consideration of a serious breach and participants who do not adhere to the assigned treatment arm due to any of (i) –(iv) will be excluded from the per-protocol analysis.

# 10. CONFIDENTIALITY

Participant confidentiality is strictly held in trust by the participating investigators, research staff, and the sponsoring institution and their agents. This confidentiality is extended to cover testing of biological samples in addition to the clinical information relating to participating participants.

To preserve confidentiality and reduce the risk of identification during collection, analysis and storage of data and information, the following will be undertaken:

(1) The number of private/confidential variables collected for each individual has been minimised. The data collected will be limited to that required to address the primary and secondary objectives.
(2) Participant identifiers will be stored separately to the data collected; documents with identifiers will be stored separately to participant data. Participant data and samples will be identified through use of a unique participant trial number/code assigned to the trial participant (“re-identifiable”). The Site Principal Investigator is responsible for the storage of a master-file of names and other identifiable data with the participant ID; access to this document will be restricted to the site trial team and authorised persons as listed previously. The master file should be stored securely, and separately, from trial data in locked/ password-protected databases with passwords kept separately.
(3) Separation of the roles responsible for management of identifiers and those responsible for analysing content. The data will be analysed by the Principal Investigator, supervised by the trial statistician, who will be provided with anonymised data identified only by the unique participant trial ID.

The trial protocol, documentation, data, and all other information generated will be held in strict confidence. No information concerning the trial, or the data will be released to any unauthorised third party, without prior written approval of the sponsoring institution. Authorised representatives of the sponsoring institution may inspect all documents and records required to be maintained by the Investigator, including but not limited to, medical records (office, clinic, or hospital) and pharmacy records for the participants in this trial. The clinical trial site will permit access to such records.

All evaluation forms, reports and other records that leave the site will be identified only by the Unique Participant Identifier (Participant ID) to maintain participant confidentiality.

Clinical information will not be released without written permission of the participant, except as necessary for monitoring by HREC or regulatory agencies.

**12.1 Data Storage**

Hard copy data will be securely stored by the participating site in a locked cupboard in a secured location. Electronic data will be securely stored on the CHOICE-UTI REDCap Data Management System (DMS), hosted by MCRI IT Department. Trial related essential documents maintained for the study will be filed within the CHOICE-UTI electronic Trial Master File (eTMF) platform, Florence eBinders™, a cloud-based SaaS software maintained by Florence Healthcare, hosted in Germany, EU via Amazon Web-Services (AWS) and backed up daily. Site Information Files (SIF) and Investigator Site Files (ISF) pertaining to each participating site will also be maintained via the Florence eBinders™ platform. Refer to the Section 8 for detailed information on data storage.

# 11. PARTICIPANT REIMBURSEMENT

Not applicable. No participant reimbursement will take place for participation in the trial.

# 12. FINANCIAL DISCLOSURE AND CONFLICTS OF INTEREST

The investigators do not have any financial or other competing interests to disclose.

# 13. DISSEMINATION AND TRANSLATION PLAN

Aggregate data will be used internally to evaluate the outcomes of each intervention arm.

It is anticipated that results from the project will be published in appropriate peer-reviewed journals.

# 14. ADDITIONAL CONSIDERATIONS

Participants who cannot understand written English will not be excluded from recruitment. The research team will describe the trial and request participation (consent) with the assistance of a certified translator. The translator will be provided by the site the patient presents to. An overview of the study will also be available on an MCRI webpage. (<https://www.mcri.edu.au/research/projects>.)

# 15. REFERENCES

1.	Winberg J, Andersen HJ, Bergstrom T, Jacobsson B, Larson H, Lincoln K. Epidemiology of symptomatic urinary tract infection in childhood. Acta Paediatr Scand Suppl. 1974(252):1-20. Epub 1974/01/01. doi: 10.1111/j.1651-2227.1974.tb05718.x. PubMed PMID: 4618418.

2.	Britt H, Miller GC, Henderson J, Bayram C, Harrison C, Valenti L, et al. General practice activity in Australia 2015-16. Sydney: Sydney University Press; 2016.

3.	NHPA. Healthy Communities: Potentially preventable hospitalisations in 2013-14. Sydney: National Health Priority Areas, 2015.

4.	Vallejo-Torres L, Pujol M, Shaw E, Wiegand I, Vigo JM, Stoddart M, et al. Cost of hospitalised patients due to complicated urinary tract infections: a retrospective observational study in countries with high prevalence of multidrug-resistant Gram-negative bacteria: the COMBACTE-MAGNET, RESCUING study. BMJ Open. 2018;8(4):e020251. Epub 2018/04/15. doi: 10.1136/bmjopen-2017-020251. PubMed PMID: 29654026; PubMed Central PMCID: PMCPMC5898316.

5.	Montini G, Toffolo A, Zucchetta P, Dall'Amico R, Gobber D, Calderan A, et al. Antibiotic treatment for pyelonephritis in children: multicentre randomised controlled non-inferiority trial. BMJ. 2007;335(7616):386. Epub 2007/07/06. doi: 10.1136/bmj.39244.692442.55. PubMed PMID: 17611232; PubMed Central PMCID: PMCPMC1955287.

6.	Neuhaus TJ, Berger C, Buechner K, Parvex P, Bischoff G, Goetschel P, et al. Randomised trial of oral versus sequential intravenous/oral cephalosporins in children with pyelonephritis. Eur J Pediatr. 2008;167(9):1037-47. Epub 2007/12/13. doi: 10.1007/s00431-007-0638-1. PubMed PMID: 18074149.

7.	Hoberman A, Wald ER, Hickey RW, Baskin M, Charron M, Majd M, et al. Oral versus initial intravenous therapy for urinary tract infections in young febrile children. Pediatrics. 1999;104(1 Pt 1):79-86. Epub 1999/07/02. doi: 10.1542/peds.104.1.79. PubMed PMID: 10390264.

8.	Bocquet N, Sergent Alaoui A, Jais JP, Gajdos V, Guigonis V, Lacour B, et al. Randomized trial of oral versus sequential IV/oral antibiotic for acute pyelonephritis in children. Pediatrics. 2012;129(2):e269-75. Epub 2012/02/01. doi: 10.1542/peds.2011-0814. PubMed PMID: 22291112.

9.	Strohmeier Y, Hodson EM, Willis NS, Webster AC, Craig JC. Antibiotics for acute pyelonephritis in children. Cochrane Database Syst Rev. 2014(7):CD003772. Epub 2014/07/30. doi: 10.1002/14651858.CD003772.pub4. PubMed PMID: 25066627.

10.	Roberts KB. Revised AAP Guideline on UTI in Febrile Infants and Young Children. Am Fam Physician. 2012;86(10):940-6. Epub 2012/11/20. PubMed PMID: 23157147.

11.	Baumer JH, Jones RW. Urinary tract infection in children, National Institute for Health and Clinical Excellence. Arch Dis Child Educ Pract Ed. 2007;92(6):189-92. Epub 2007/11/23. doi: 10.1136/adc.2007.130799. PubMed PMID: 18032715.

12.	Basmaci R, Vazouras K, Bielicki J, Folgori L, Hsia Y, Zaoutis T, et al. Urinary Tract Infection Antibiotic Trial Study Design: A Systematic Review. Pediatrics. 2017;140(6). Epub 2017/12/01. doi: 10.1542/peds.2017-2209. PubMed PMID: 29187579.

13.	Ahmed TA. Pharmacokinetics of drugs following IV bolus, IV infusion, and oral administration. In: Ahmed TA, editor. Basic Pharmacokinetic Concepts and Some Clinical Applications. London: InTech; 2015.

14.	Goodlet KJ, Benhalima FZ, Nailor MD. A Systematic Review of Single-Dose Aminoglycoside Therapy for Urinary Tract Infection: Is It Time To Resurrect an Old Strategy? Antimicrob Agents Chemother. 2019;63(1). doi: 10.1128/AAC.02165-18. PubMed PMID: 30397061; PubMed Central PMCID: PMCPMC6325212.

15.	Grimwood K, Abbott GD, Fergusson DM. Single dose gentamicin treatment of urinary infections in children. N Z Med J. 1988;101(852):539-41. Epub 1988/08/24. PubMed PMID: 3045718.

16.	Khan AJ, Kumar K, Evans HE. Single-dose gentamicin therapy of recurrent urinary tract infection in patients with normal urinary tracts. J Pediatr. 1987;110(1):131-5. Epub 1987/01/01. doi: 10.1016/s0022-3476(87)80308-6. PubMed PMID: 3794874.

17.	Scanlan BT, Ibrahim OF, Hopper SM, McNab S, Babl FE, Davidson A, et al. The Where and the Why of Intravenous Antibiotic Treatment for Febrile Urinary Tract Infection/Pyelonephritis in Children. European Academyof Paediatrics Society Meeting; Ljubljana, Slovenia2017.

18.	Scanlan BT, Ibrahim LF, Hopper SM, Babl FE, Davidson A, Bryant PA. Selected Children With Complicated Acute Urinary Tract Infection May Be Treated With Outpatient Parenteral Antibiotic Therapy at Home Directly From the Emergency Department. Pediatr Infect Dis J. 2019;38(2):e20-e5. Epub 2018/04/11. doi: 10.1097/INF.0000000000002070. PubMed PMID: 29634622.

19.	Scanlan B, Ibrahim L, Hopper S, McNab S, Donath S, Babl F, et al. G122(P) Intravenous or oral antibiotics for urinary tract infection/pyelonephritis in children? Development of the melbourne rupert score. Archives of Disease in Childhood. 2019;104(Suppl 2):A49-A50. doi: 10.1136/archdischild-2019-rcpch.118.

20.	Duggan A, Koff E, Marshall V. Clinical variation: why it matters. Med J Aust. 2016;205(10):S3-S4. Epub 2016/11/18. doi: 10.5694/mja16.00819. PubMed PMID: 27852193.

21.	Ibrahim LF, Hopper SM, Connell TG, Daley AJ, Bryant PA, Babl FE. Evaluating an admission avoidance pathway for children in the emergency department: outpatient intravenous antibiotics for moderate/severe cellulitis. Emerg Med J. 2017;34(12):780-5. Epub 2017/10/06. doi: 10.1136/emermed-2017-206829. PubMed PMID: 28978652.

22.	Ibrahim LF, Hopper SM, Orsini F, Daley AJ, Babl FE, Bryant PA. Efficacy and safety of intravenous ceftriaxone at home versus intravenous flucloxacillin in hospital for children with cellulitis (CHOICE): a single-centre, open-label, randomised, controlled, non-inferiority trial. Lancet Infect Dis. 2019;19(5):477-86. Epub 2019/03/12. doi: 10.1016/S1473-3099(18)30729-1. PubMed PMID: 30853250.

23.	Ibrahim LF, Hopper SM, Babl FE, Bryant PA. Who Can Have Parenteral Antibiotics at Home?: A Prospective Observational Study in Children with Moderate/Severe Cellulitis. Pediatr Infect Dis J. 2016;35(3):269-74. Epub 2015/11/17. doi: 10.1097/INF.0000000000000992. PubMed PMID: 26569189.

24.	Ibrahim LF, Huang L, Hopper SM, Dalziel K, Babl FE, Bryant PA. Intravenous ceftriaxone at home versus intravenous flucloxacillin in hospital for children with cellulitis: a cost-effectiveness analysis. Lancet Infect Dis. 2019;19(10):1101-8. Epub 2019/08/20. doi: 10.1016/S1473-3099(19)30288-9. PubMed PMID: 31420292.

25.	ACSQHC. AURA 2017: second Australian report on antimicrobial use and resistance in human health. Sydney: Australian Commission on Safety and Quality in Health Care 2017.

26.	WHO. Ten threats to global health in 2019. World Health Organisation; 2019. p. <https://www.who.int/news-room/feature-stories/ten-threats-to-global-health-in-2019>.

27.	Chelimo C, Camargo CA, Jr., Morton SMB, Grant CC. Association of Repeated Antibiotic Exposure Up to Age 4 Years With Body Mass at Age 4.5 Years. JAMA Netw Open. 2020;3(1):e1917577. Epub 2020/01/23. doi: 10.1001/jamanetworkopen.2019.17577. PubMed PMID: 31968112.

28.	The Royal Children's Hospital. Clinical Practice Guideline on Urinary Tract Infection [Internet, last updated November 2017; cited 20 August 2019]. Available from: www.rch.org.au/clinicalguide/index.cfm.

29.	Okarska-Napierala M, Wasilewska A, Kuchar E. Urinary tract infection in children: Diagnosis, treatment, imaging - Comparison of current guidelines. J Pediatr Urol. 2017;13(6):567-73. Epub 2017/10/08. doi: 10.1016/j.jpurol.2017.07.018. PubMed PMID: 28986090.

30.	McMullan BJ, Andresen D, Blyth CC, Avent ML, Bowen AC, Britton PN, et al. Antibiotic duration and timing of the switch from intravenous to oral route for bacterial infections in children: systematic review and guidelines. Lancet Infect Dis. 2016;16(8):e139-52. doi: 10.1016/S1473-3099(16)30024-X. PubMed PMID: 27321363.

31.	Daniel M, Szajewska H, Panczyk-Tomaszewska M. 7-day compared with 10-day antibiotic treatment for febrile urinary tract infections in children: protocol of a randomised controlled trial. BMJ Open. 2018;8(3):e019479. Epub 2018/03/04. doi: 10.1136/bmjopen-2017-019479. PubMed PMID: 29500209; PubMed Central PMCID: PMCPMC5855174.
